# Genome-Wide Investigation of MicroRNAs and Their Targets in Response to Freezing Stress in *Medicago sativa* L., Based on High-Throughput Sequencing

**DOI:** 10.1534/g3.115.025981

**Published:** 2016-01-20

**Authors:** Yongjun Shu, Ying Liu, Wei Li, Lili Song, Jun Zhang, Changhong Guo

**Affiliations:** Key Laboratory of Molecular Cytogenetics and Genetic Breeding of Heilongjiang Province, College of Life Science and Technology, Harbin Normal University, 150025, China

**Keywords:** *Medicago sativa*, cold acclimation, freezing stress, microRNA, degradome sequencing

## Abstract

Winter damage, especially in northern climates, is a major limitation of the utilization of perennial forages such as alfalfa. Therefore, improving freezing tolerance is imperative in alfalfa genetic breeding. However, freezing tolerance is a complex trait that is determined by many genes. To understand the complex regulation mechanisms of freezing tolerance in alfalfa, we performed small RNA sequencing analysis under cold (4°) and freezing (−8°) stress. The sequencing results revealed that 173 known, and 24 novel miRNAs were expressed, and that the expression of 35 miRNAs was affected by cold and/or freezing stress. Meanwhile, 105 target genes cleaved by these miRNAs were characterized by degradome sequencing. These targets were associated with biological regulation, cellular processes, metabolic processes, and response to stress. Interestingly, most of them were characterized as transcription factors (TFs), including auxin response factors, SBP, NAC, AP2/ERF, and GRF, which play important roles in plant abiotic responses. In addition, important miRNAs and mRNAs involved in nodulation were also identified, for example, the relationship between miR169 and the TF CCAAT (also named as NF-YA/HAP2), which suggested that nodulation has an important function in freezing tolerance in alfalfa. Our results provide valuable information to help determine the molecular mechanisms of freezing tolerance in alfalfa, which will aid the application of these miRNAs and their targets in the improvement of freezing tolerance in alfalfa and related plants.

Lack of tolerance to freezing is a major environmental limitation of the survival, productivity, and ecological distribution of plants. However, freezing tolerance is a complex trait that is determined by numerous factors from plants and the environment. Among these factors, cold acclimation, the exposure of plants to low, subfreezing temperatures, plays an important role in conferring freezing tolerance (Thomashow 2010). During the cold acclimation process, several biochemical and physiological modifications occur, including accumulation of soluble sugars, free amino acids, and the expression of cold-regulated (COR) genes, which potentially improving freezing tolerance in plants. To date, the identification and characterization of C-repeat (CRT)-binding factors (CBFs) has shown that they play critical roles in the cold acclimation process (Gilmour *et al.* 1998; Thomashow 2010), by regulating downstream functional genes, such as COR genes (Hajela *et al.* 1990; Thomashow 2010). Regulation of COR genes by CBFs constitutes the central component of cold signaling pathways that confer freezing tolerance on plants. In addition, the CBF signaling pathway is also regulated by other factors, for example, ICE1 (Inducer of CBF Expression 1) (Chinnusamy *et al.* 2003; Lee *et al.* 2005; Miura and Hasegawa 2008), and HOS1 (High Expression of Osmotically Responsive Gene 1) (Jung and Park 2013; Lee *et al.* 2001), which are important components in the plant cold acclimation process.

MicroRNAs are a class of noncoding small RNAs of approximately 21–24 nt, which bind to complementary sequences in the mRNAs of target genes (Mallory and Vaucheret 2006). This binding results in the regulation of gene expression at the posttranscriptional level by cleavage-induced degradation of the mRNA, or suppression of its translation (Mallory and Vaucheret 2006). In recent years, many miRNAs have been demonstrated to have important regulatory functions in plant growth, development, and stress responses (Babar *et al.* 2008; Khraiwesh *et al.* 2012; Sunkar *et al.* 2012). A number of miRNAs are involved in the cold response process in *Arabidopsis* (Liu *et al.* 2008; Sunkar and Zhu 2004; X. Zhou *et al.* 2008), poplar (Chen *et al.* 2012; Lu *et al.* 2008), *Brachypodium distachyon* (Zhang *et al.* 2009), rice (Lv *et al.* 2010), and wheat (Tang *et al.* 2011, 2012), including miR156/157, miR169, miR393, miR396, miR394, and miR398 (Rajwanshi *et al.* 2014). With the development of high-throughput sequencing technology, numerous miRNAs have been identified and characterized in plants. MiRNAs biological functions have been deduced by the identification and characterization of their target genes. Degradome sequencing, also termed parallel analysis of RNA ends (PARE), was developed based on high-throughput sequencing technology for the genome-wide identification of miRNA target genes (Folkes *et al.* 2012). Using high throughput sequencing methods, many new miRNA–mRNA target pairs have been identified successfully in *Arabidopsis* (Addo-Quaye *et al.* 2008), rice (Sun *et al.* 2015), wheat (Chen *et al.* 2015a), soybean (Shamimuzzaman and Vodkin 2012), maize (Zhao *et al.* 2013), and grapevine (Pantaleo *et al.* 2010), which has helped lucidate the regulatory relationships between miRNAs and their target genes.

Alfalfa (*Medicago sativa* L.), which is grown worldwide, is a highly productive perennial forage species with the capacity for biological fixation of atmospheric nitrogen. However, because of insufficient freezing tolerance, hard winters (with extremely low temperatures) are a major limitation to alfalfa production (Castonguay *et al.* 2013; Pennycooke *et al.* 2008). Thus, improvement of freezing tolerance is an important breeding aim for high yield and longer production periods in alfalfa, especially in northern climates, for example, in the United States, Canada, and China (Chen *et al.* 2015b). *M. sativa* L. cv. Zhaodong was domesticated and bred from wild *M. sativa* by the Institute of Animal Husbandry of Heilongjiang Province (IAH-HLJ, China), and has high tolerance to freezing stress, enabling it to survive during the winter season in the fields of Heilongjiang Province, China (average temperature –35°). The high freezing tolerance of *M. sativa* L. cv. Zhaodong is determined by its specific gene regulation network (Luo *et al.* 2004). Determining the specific expression patterns of miRNAs and mRNAs would be helpful to understand the complex molecular mechanism of freezing tolerance in this species.

In the present study, miRNAs and their targets that are involved in cold and/or freezing stress were investigated using high-throughput sequencing. Freezing-stress-responsive miRNAs were selected and validated by quantitative real-time reverse transcription (qRT-PCR) experiments. Meanwhile, the potential miRNA targets were predicted and confirmed by degradome sequencing.

## Materials and Methods

### Plant growth and treatment

Seeds of *M. sativa* (cv. Zhaodong), kindly provided by Prof. Hong Li (IAH-HLJ, China), were germinated and transferred onto a mix of perlite and sand (3:1, v/v). All seedlings were grown in a growth chamber (Conviron E15, Canada), at a temperature between 18° (night) and 24° (day), with humidity ranging from 60% to 80%, and a light period of 14 hr/10 hr (daytime, 06:00–20:00). The seedlings were irrigated with half-strength Hoagland solution once every other day, and after 8 wk they were randomly divided into three groups for stress treatments. For the control group (untreated, A group), the seedlings continued to grow at 18° (night) to 24° (day). For cold stress (B group) and freezing stress (C group), the seedlings were transferred into another chamber with the temperature set at 4° or −8°, respectively. According to our previous research (Shu *et al.* 2015), all seedlings were harvested at 3 hr after stress treatments; five whole seedlings from each group were bulked separately. All samples were frozen in liquid nitrogen, and stored at −80° until use.

### Small RNA library construction and sequencing

Small RNAs were extracted from samples from the three treatments (control, cold, and freezing) using the TRIzol method (Invitrogen, Carlsbad, CA), according to the manufacturer’s instructions. Small RNAs were ligated sequentially to 5′- and 3′- RNA/DNA chimeric oligonucleotide adaptors; the resulting ligation products were gel-purified by 15% denaturing PAGE, and reverse-transcribed to produce cDNAs. The cDNAs were sequenced using a Genome Analyzer IIx System, according to the manufacturer’s instructions (BGI-Shenzhen Co. Ltd., Shenzhen, China).

### Identification of conserved and novel miRNAs

Raw data were first processed by filtering out low-quality reads, trimming the adaptors, and removing other noise reads, to obtain clean reads. The clean reads were then aligned to the Rfam database to remove other noncoding RNAs, including rRNA, tRNA, and snRNA. The remaining reads were mapped to assembled transcriptome sequences from *M. sativa* cv. Zhaodong (as described in the section below on transcriptome sequencing, *RNA-seq library construction*, *sequencing and analysis*). Mapping reads were retrieved and aligned to plant miRNA sequences from miRBase V21 (Kozomara and Griffiths-Jones 2014) using Bowtie (Langmead and Salzberg 2012), which identified and annotated conserved miRNA genes. To identify novel miRNA genes, first, miRDeep-P retrieved the flanking assembled transcriptome sequences of mapping reads, which were identified as candidate precursors of miRNA (Yang and Li 2011). Second, based on the precursors, the novel miRNAs were identified using MIREAP, and their secondary structures were verified by the software RNAfold, as previously reported (Denman 1993). In addition, plant miRNA criteria mentioned for Arabidopsis (Meyers *et al.* 2008), and rice (Jeong *et al.* 2011), were also considered for alfalfa novel miRNA identification.

All miRNA abundances were evaluated and normalized using the tags per million reads (TPM) method, based on BLAST mapping results. The TPM values were calculated as follows: TPM = number of mapped miRNA reads × 10^6^ / number of clean sample reads. The normalized expression was adjusted to 0.01 when miRNA expression (TPM) was zero to avoid negating the subsequent calculation of fold change. The miRNA expression fold changes between stress and control groups (Cold/Control, Freezing/Control) were computed, and chi-squared tests were performed to determine the significance of miRNA expression for each comparison using the R software. The miRNAs with fold change (TPM ratios) ≥ 2 or ≤ 0.5, and p-value ≤ 0.05 were deemed differentially expressed in response to cold and/or freezing stresses, as described by Xie *et al.* (2014).

### Quantitative real-time reverse transcription PCR (qRT-PCR) analysis of miRNA expression

The expression of 12 selected miRNAs from the three conditions were assayed using stem-loop quantitative reverse transcription PCR (qRT-PCR). Primers for all miRNAs, and the reference gene (U6), were designed as shown in Supporting Information, Table S1. Total RNA was extracted from alfalfa grown under the three conditions (control, cold, and freezing) with TRIZOL reagent, according to the manufacturer’s instructions (Invitrogen). These RNAs were reverse transcribed to cDNA, according to the manufacturer’s protocol. qRT-PCR was performed using the LightCycler 480II Detection System (Roche) with a total reaction volume of 20 μl, containing 1 μl of cDNA templates, 8 μM of primer mix, 10.0 μl of 2 × SYBR Green Mix, and 8.7 μl ddH_2_O. The PCR conditions were set as follows: 95° for 2 min; 40 cycles of 95° for 10 sec, 60° for 30 sec, and 60° for 45 sec. The miRNA expression abundances were determined based on the 2^–ΔΔCT^ method, and relative changes in miRNA expression from the qRT-PCR experiments were calculated. Three biological replicates for each group were run, and each reaction was performed with three technical replicates.

### Degradome library construction and target identification

To investigate the potential target mRNAs, two degradome libraries from cold stress and freezing stress samples were constructed, as previously described (Folkes *et al.* 2012). In brief, poly(A)-enriched RNAs were isolated, and ligated to an RNA oligo-nucleotide adaptor containing a 3′-*Mme*I recognition site; the ligated products were used to synthesize first-strand cDNA. A short PCR (five cycles) reaction was used to amplify the cDNA, and the product was ligated to a double-stranded DNA adaptor, before being subjected to gel purification again for PCR amplification. The final cDNA library was purified and sequenced on an Illumina GAII by BGI-Shenzhen Co. Ltd (Shenzhen, China).

Adaptor sequences and low quality sequencing reads were removed from the raw reads, and the clean reads were used to identify potentially cleaved targets based on *M. sativa* assembled transcriptome sequences (described below) by the CleaveLand4 pipeline (Addo-Quaye *et al.* 2009). Meanwhile, the psRNATarget tool was also used to predict miRNA targets using a set of default parameters (Dai and Zhao 2011). MiRNA target genes were also predicted from *M. sativa* assembled transcriptome sequences. Prediction targets were used to cross-check the degradome sequencing results.

### RNA-seq library construction, sequencing, and analysis

Total RNAs were extracted from three samples using the RNeasy Plant Mini Kit (Qiagen, Valencia, CA), and transcriptome sequencing libraries were constructed according to the manufacturer’s instructions. In brief, short fragments were purified using a MinElute PCR Purification Kit (Qiagen), and eluted in 10 μl of EB buffer (Qiagen). The short fragments were ligated with sequencing adapters, and the desired fragments (around 250 bp) were separated by agarose gel electrophoresis, and purified using a gel extraction kit. Finally, the sequencing library was constructed by linear PCR amplification (15 cycles), and sequenced using the Illumina GAII platform by BGI-Shenzhen Co. Ltd (Shenzhen, China), generating 100-bp pair-end reads. Processing of raw data, removal of adapter sequences, base-calling, and quality value calculations, were performed to produce clean data. Clean reads from three libraries were assembled *de novo* into contigs using Trinity software with the following parameter: “min_kmer_cov 2” (Haas *et al.* 2013). To remove redundancy among the Trinity-generated contigs, they were further assembled *de novo* using iAssembler, with the minimum percent identify (−p) set to 97 (Zheng *et al.* 2011). The resulting unique transcripts were identified as *M. sativa* transcriptome unique assembled mRNA sequences. These assembled transcripts were BLAST-searched against combined databases of Arabidopsis, rice, soybean, and *Medicago truncatula* protein sequences for functional annotation (the e-value was set at 1E-5). Gene ontology (GO) annotations were assigned to the targets based on the GO terms annotated to their corresponding homologs in the combined database, and the GO enrichment analysis of miRNA targets was performed using package topGO on the R platform. Meanwhile, plant transcription factors (TFs) from *M. sativa* were identified and classified into different families using the iTAK pipeline (http://bioinfo.bti.cornell.edu/tool/itak) (Jin *et al.* 2014).

Clean reads from the three samples were mapped to the *M. sativa* assembled transcripts generated by RNA-seq, as previously described, using the TopHat software (Trapnell *et al.* 2009), and mRNA target gene expressions [as estimated by the fragments per kilobase of exon per million fragments mapped (FPKM) method] across the control, cold, and freezing samples were evaluated using the Cufflinks software (Trapnell *et al.* 2012). Differential expression analysis was performed using the edgeR package (Robinson *et al.* 2010) on the R platform, and target genes with fold changes ≥ 2 or ≤ 0.5, with an adjusted p-value ≤ 0.01, were identified as differentially expressed in response to cold, and/or freezing stress.

### Data availability

The *Medicago sativa* (cv. Zhaodong) small RNA sequences, degradome sequences, and transcriptome sequences are all deposited into NCBI SRA with accession number: SRP064230.

## Results

### High-throughput sequencing of small RNA libraries

Raw reads of three libraries were obtained by high-through sequencing, and were deposited with NCBI SRA under accession number SRP064230. After removing low-quality reads, poly(A) reads, oversized insertions, reads shorter than 18 nt, and adaptor-contaminated reads, 10,823,011, 10,833,023, and 10,781,132 clean reads were generated from the control, cold, and freezing libraries, respectively. These reads included unique reads, and the length distribution of small RNA reads from the three libraries ranged from 18 nt to 28 nt (see Figure S1). The majority of small RNA reads were 20–24 nt sequences, comprising over 80% of the reads. The 21 nt and 24 nt small RNAs comprised the two major classes, which was consistent with previous publications (Fan *et al.* 2014; Tang *et al.* 2012; Zhang *et al.* 2009). Other noncoding RNAs, including rRNA, tRNA, and snRNA, were removed by mapping reads to the Rfam database. The remaining reads were mapped to the alfalfa transcriptome assembled sequences; about 41% of the reads were mappable (Table 1). To identify conserved miRNAs in alfalfa, the mappable reads were aligned to known plant miRNAs in the miRBase database, using Bowtie with no more than one mismatch. In total, 1,597,215, 1,287,494, and 987,515 reads were identified as homologous to known miRNAs from the control, cold, and freezing libraries, respectively. They were identified as 173 conserved miRNA genes from 112 miRNA families (see Table S2). The flanking sequences of the remaining mappable reads were retrieved and analyzed by MIREAP using plant default parameters. In total, 24 novel miRNAs among the predicted RNA hairpins were identified in alfalfa, most of them were 21 nt and 24 nt in length (Table S3).

**Table 1:** Overview of small RNA sequences in three alfalfa libraries

Data Type	Control		Cold		Freezing	
	Total Reads	Unique Reads	Total Reads	Unique Reads	Total Reads	Unique Reads
Clean reads	10,823,011	4,415,425	10,833,023	4,746,716	10,781,132	4,296,444
Rfam	229,925	18,696	400,604	28,495	1,947,224	38,460
Transcriptome	4,933,169	1,201,107	4,509,305	1,232,313	3,892,472	1,219,439
Known miRNA	1,597,215	24,334	1,287,494	27,292	987,515	24,278
Novel miRNA	172,273	1355	82,573	1121	62,290	944

To identify miRNAs involved in alfalfa response to cold and freezing stress, the miRNA expressions of three groups were evaluated and normalized (see Table S4). Compared with the control group, miRNAs with a log2 fold change higher than 1, or less than –1, combined with a p-value less than 0.05, were identified as differentially expressed miRNAs. There were 35 differentially expressed miRNAs in response to cold, and/or freezing stress (Table 2). Among these miRNAs, 12 were regulated by cold stress, while 30 responded to freezing stress. Nine miRNAs responded to cold and freezing stress, three miRNAs were specifically regulated by cold stress, and 23 miRNAs specifically responded to freezing stress. In addition, most (29 miRNAs) were downregulated by cold and/or freezing stress; only six miRNAs were induced by cold and/or freezing stress.

**Table 2:** The differential expression of miRNA genes in alfalfa in response to cold and/or freezing stresses

miRNA	Fold Change		Statistical Significance	
	Cold	Freezing	Cold	Freezing
miR156c-3p	−0.62	−1.02	**	**
miR156i-5p	−0.78	−1.43	**	**
miR159a	−1.85	−1.15	**	**
miR159b	0.00	9.80		**
miR160e	0.11	1.50		**
miR166e-5p	−0.58	−1.64		**
miR166f	1.52	2.81	**	**
miR166g-5p	−0.75	−1.97	**	**
miR167a	−1.11	−1.53	**	**
miR167b-5p	−0.94	−1.99	**	**
miR168c-3p	−0.19	−1.04		**
miR171a	−0.99	1.43		**
miR172a	−1.77	−2.30	**	**
miR172c-3p	−1.21	−1.82	**	**
miR172d-3p	−1.31	−0.69	**	**
miR2119	−0.90	2.08		**
miR396a-5p	−1.74	−2.31	**	**
miR396b-3p	−0.71	−1.16	*	**
miR398a-5p	−2.28	0.17	**	
miR5037c	2.01	1.63	**	*
miR5231	−0.93	−1.94	**	**
miR5232	0.10	−1.01		*
miR5234	−0.63	−1.37	*	**
miR5239	−0.85	−1.58	**	**
miR5287b	−0.30	−1.83		**
NmiR0018	−1.01	−0.28	**	*
NmiR0019	−0.62	−3.60	**	**
NmiR0026	−0.69	−1.37	**	**
NmiR0028	−0.84	−1.07	**	**
NmiR0029	−0.98	−1.85	**	**
NmiR0043	−0.51	−1.92		**
NmiR0047	−0.45	−2.18	*	**
NmiR0049	−1.79	−2.58	**	**
NmiR0051	−2.04	−2.21	**	**
NmiR0053	−0.52	−2.20		*

The miRNA abundance was evaluated and normalized using the TPM method, and the expression values of miRNAs whose expression was zero were adjusted to 0.01. The fold changes were computed from cold [log_2_(Cold/Control)] and freezing [log_2_(Freezing/Control)] based expression. Meanwhile, chi-squared tests were also performed to test the significance of each comparison, using the R software. A p-value no more than 0.01 was set as the extremely significant level (**), while a p-value greater than 0.01 and no more than 0.05 was defined as significant (*).

### qRT-PCR validation of miRNA expression

**Figure 1:**
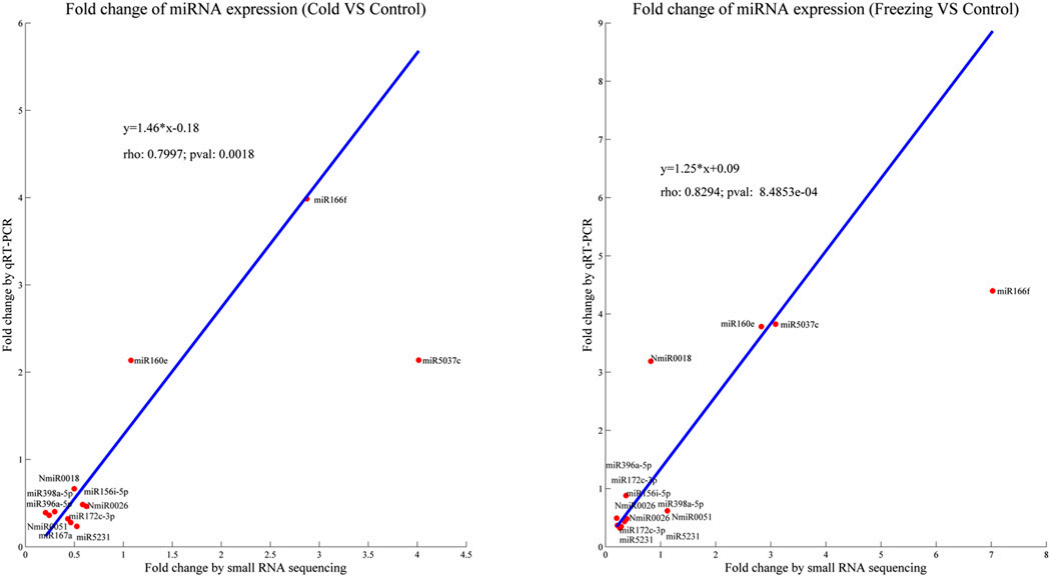
Comparison of the expression of 12 miRNAs between small RNA sequencing and qRT-PCR in response to cold and freezing stress. Red dots are plot-based fold changes of each miRNA gene between the abundance from RNA sequencing and qRT-PCR detection. The line correction relationship was computed based on the expression of 12 miRNA genes (blue line).

**Figure 2:**
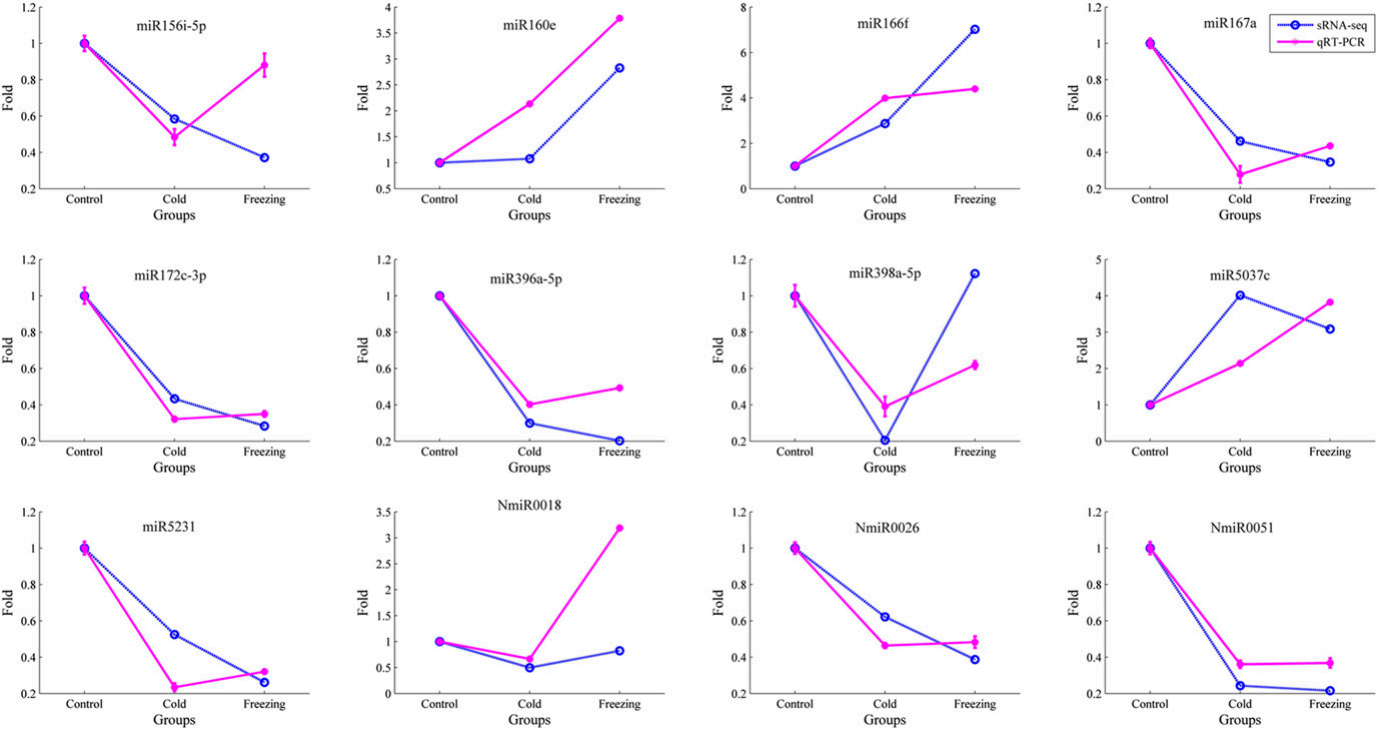
Validation of the expression of 12 miRNAs in alfalfa using qRT-PCR. Expressional abundance of each miRNA gene in the control sample was set as 1, and fold changes of each miRNA gene relative to the control sample were calculated. Fold change values greater than 2 or less than 0.5 indicate upregulated or downregulated miRNAs. The blue plots are small RNA sequencing results, and pink represents the qRT-PCR results.

To validate an miRNAs’ function in alfalfa in response to cold and freezing stress, 12 differentially expressed miRNAs were selected for qRT-PCR detection. The means of the correlation coefficients of the qRT-PCR validations, and the high-throughput sequencing results for the miRNAs were as high as 0.80 and 0.83 under cold and freezing stress, respectively, which implied that our miRNA sequencing results were highly reliable (Figure 1). For example, miR167a, miR172c-3p, miR396a-5p, and miR5231 were identified as downregulated by both cold and freezing stress by Illumina sequencing, and qRT-PCR showed the same expression profile in response to cold and freezing stress (Figure 2). Similarly, the qRT-PCR results also validated the expression patterns of miR160e and miR166f, which were upregulated by cold/freezing stress. Notably, the expression of three novel miRNAs (NmiR0018, NmiR0026, and NmiR0051) were characterized and confirmed by qRT-PCR detection. However, there were some miRNAs that showed qRT-PCR results that were inconsistent with the sequencing results, *i.e.*, miR156i-5p, miR398a-5p, and miR5037c. This possibly reflected the different sensitivities of high-throughput sequencing and the qRT-PCR detection method for specific miRNAs.

### Analysis of alfalfa transcriptome sequences

Three transcriptome libraries were sequenced, and 19,808,866, 23,870,694, 22,350,578 reads were collected from the control, cold, and freezing samples, respectively. After discarding the low-quality raw reads, the remaining reads were assembled *de novo* using the Trinity software. We obtained 124,821 assembled transcripts, with an N50 of 1392 bp, and an average length of 828 bp; detailed information is provided in Table 3. The assembled alfalfa transcripts were annotated by BLASTX analysis against Arabidopsis, rice, soybean, and *M. truncatula* proteins, revealing 73,993 (59.3%) transcripts with significant hits. The results showed that the percentage of genes that could be annotated was positively correlated with the length of the genes, as shown in Figure S2. In addition, a BLASTN search was performed against the Mt4.0v1 mRNAs (http://jcvi.org/medicago/) to estimate possible differences in transcript sequences between *Medicago sativa* and the model plant *Medicago truncatula*. The results showed that 54.1% (67,492/124,821) of assembled alfalfa transcripts had significant matches with *M. truncatula* transcripts, most of them having high identity percentages, as shown in Figure S3, which indicated high genetic similarity between the two species.

**Table 3:** Summary of the *de novo* assembled alfalfa transcriptome

Data Type	Number
Total sequence	124,821
Number of sequences in 200–500 bp	64,650
Number of sequences in 500–1000 bp	26,072
Number of sequences more than 1000 bp	34,099
Minimal length (bp)	201
Maximal length (bp)	14,177
N50 (bp)	1392
Average length (bp)	828

### Identification of miRNA targets in alfalfa

**Figure 3:**
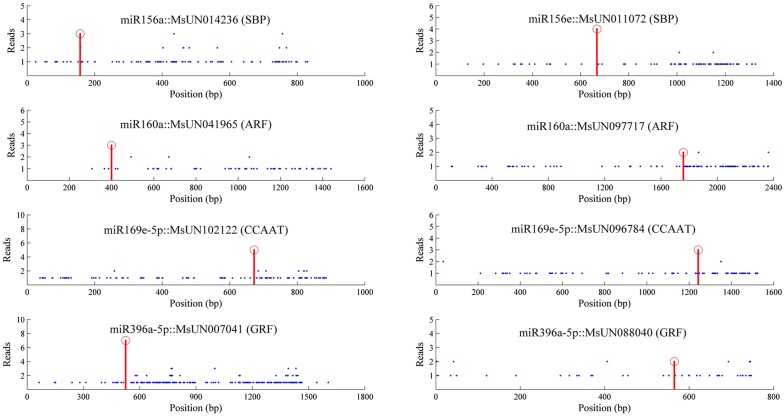
Target plots (t-plots) of miRNA targets identified by degradome sequencing in alfalfa. The values of the Reads axis indicate signature abundances of cleavage sites. Red circles on the Position axis indicate predicted cleavage sites, and red lines indicate signatures produced by miRNA-directed cleavage-based analysis by the CleaveLand4 software.

**Figure 4:**
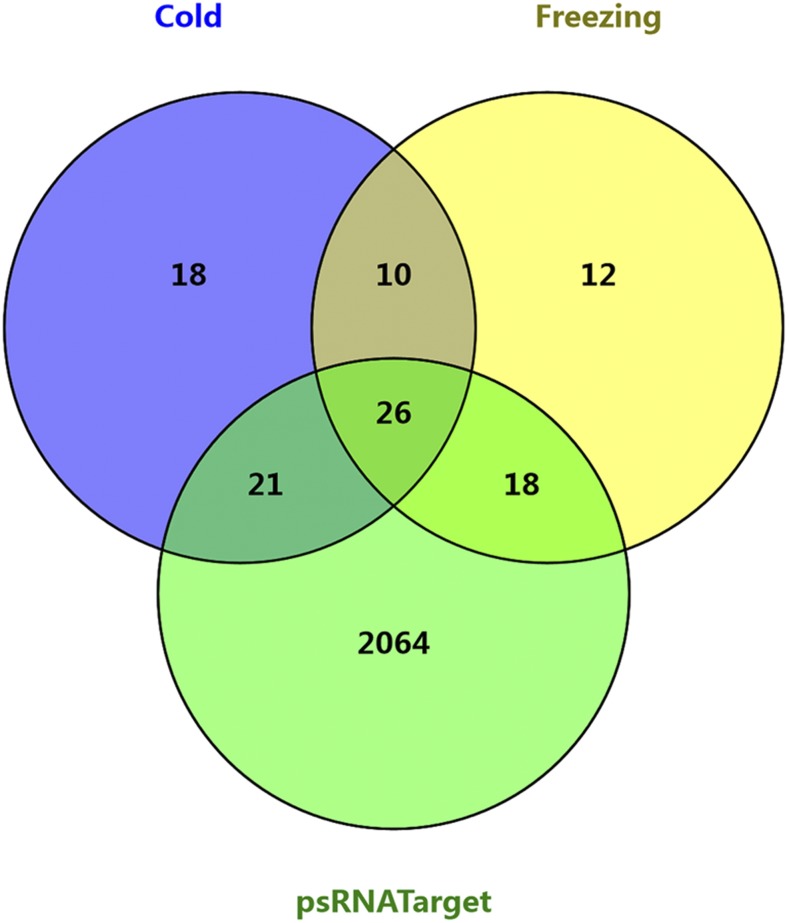
Distribution of targets genes identified by two degradome sequencing libraries, and psRNATarget. Target genes of miRNAs from cold and freezing libraries were identified by degradome sequencing, while target genes of psRNATarget were mined by scanning assembled alfalfa transcripts using psRNATarget software with default parameters.

To determine the function of miRNAs in alfalfa, degradome sequencing was used to identify the miRNA targets. After removing reads without adaptor sequences, 10,878,175 and 10,849,379 clean reads were obtained from the two degradome libraries, respectively (cold and freezing libraries); most of them were 20 nt or 21 nt in length (Figure S4). The reads were aligned to the alfalfa transcriptome sequences: 3,008,989 and 2,441,179 reads mapped to the assembled transcriptome sequences, respectively. The CleaveLand pipeline was used for further analysis, and 105 target mRNAs that were potentially cleaved by known miRNAs, and novel miRNAs, were identified from the two degradome libraries (Figure 3, File S1, File S2, and Table S5). Among these genes, 75 targets were identified in the cold stress library, 66 targets were present in freezing stress, and 36 targets were cleaved during both cold and freezing stress (Figure 4). According to the relative abundance of reads at the target sites, they were classified into five categories, 0–4, (see Figure S5). Category 0 has more than one raw read at the position, and the abundance at the position is equal to the maximum on the transcript, only one maximum. Category 1 has a similar definition to Category 0, but there is more than one maximum on the transcript. Category 2 has more than one raw read at the position. The abundance at the position is less than the maximum, but higher than the median for the transcript. Category 3 has more than one raw read at the position. The abundance at the position is equal to, or less than, the median for the transcript. Category 4 has only one raw read at the position. In this study, Category 0 had the most members under both cold and freezing stress, providing high confidence in the degradome sequencing results. To validate target genes from degradome sequencing, miRNA and transcripts were submitted to psRNATarget to identify cleavage using default parameters. There were 65 (65/105, 61.9%) targets from the degradome that were present in the psRNATarget results list (Figure 4). The results indicated that degradome sequencing was an effective method to identify cleaved targets of miRNAs.

### GO analysis and expression analysis of target genes

**Figure 5:**
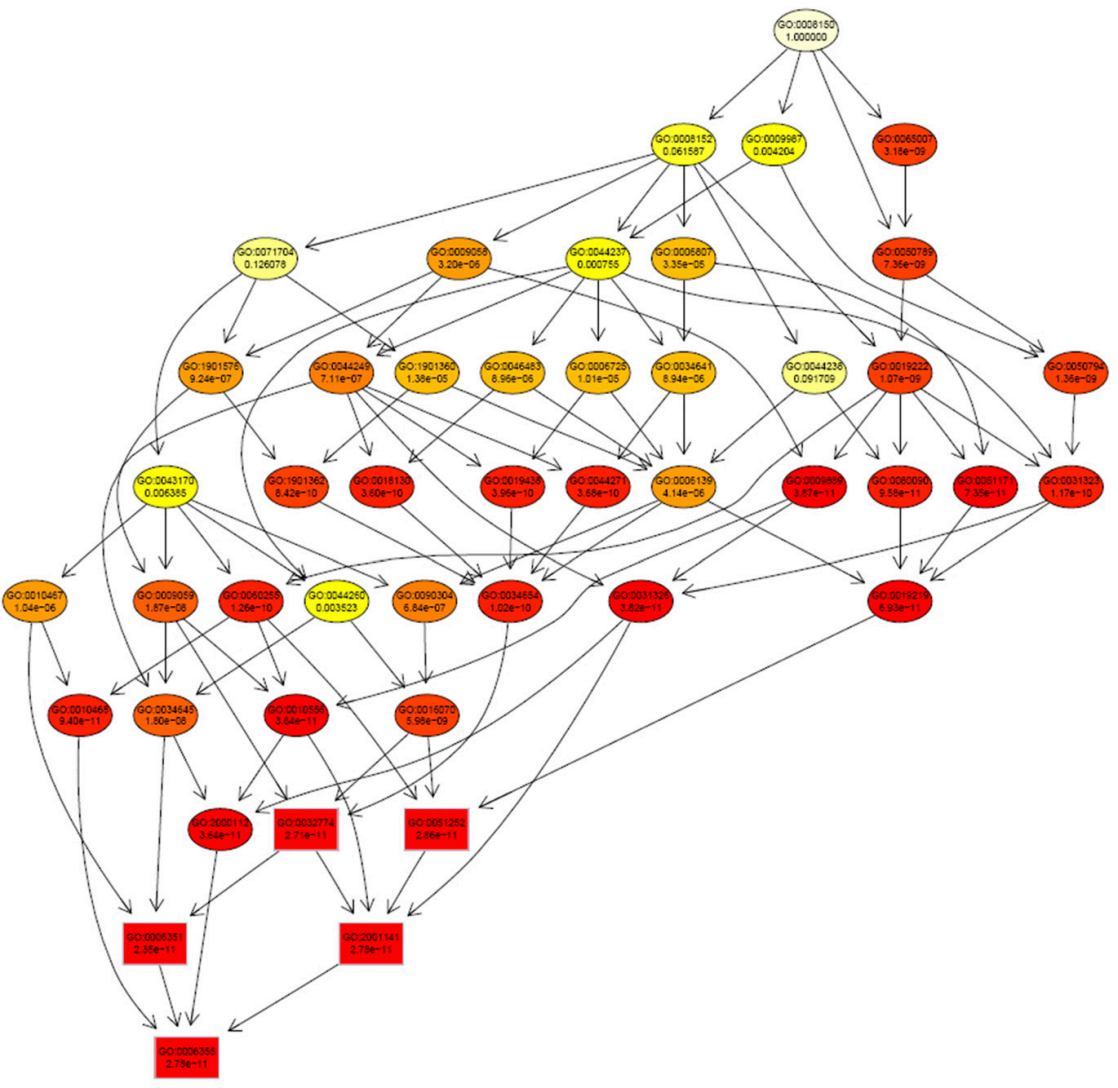
Enrichment analysis results of biological processes involving miRNA target genes in alfalfa. Biological processes were scanned by Fisher’s test using the topGO package, and each GO term was shown in yellow or red according to the p-value (yellow for a high p-value; red for a low p-value). The rectangular boxes represent the top five significant GO terms.

To further understand the functions of miRNAs, the target genes were BLAST searched against a combined database of Arabidopsis, rice, soybean, and *M. truncatula*. In total, 94 target genes were identified as homologous to functional genes from model plants, and enrichment analysis of GO annotation was performed using the topGO package (Figure 5 and Table S6). As expected, GO terms in the biological process category were highly enriched, including GO:0006355 (regulation of transcription), GO:0031323 (regulation of cellular metabolic process), GO:0050896 (response to stimulus), and GO:0009725 (response to hormone). The most enriched molecular function was GO:0003677 (DNA binding), which is consistent with the results in biological process. Similarly, the most enriched cellular components were GO:0005634 (nucleus), GO:0044424 (intracellular part), and GO:0044464 (cell part). These results implied the possible function of miRNAs in the regulation of transcription during cold and freezing stresses. To investigate transcriptional regulation in detail, we used the iTAK pipeline to scan for TFs: we identified 28 TFs as miRNA target genes (Table 4) that were highly enriched (hypergeometric test, p-value is 6.7E-20). The results were consistent with, and confirmed the results of, the GO enrichment analysis.

**Table 4:** TF targets of miRNAs identified by degradome sequencing

miRNA Family	Target Gene	TF Family
miR156	MsUN014236	SBP
	MsUN046666	SBP
	MsUN050762	SBP
	MsUN011072	SBP
	MsUN049469	SBP
	MsUN086910	SBP
miR160	MsUN041965	ARF
	MsUN097717	ARF
miR164	MsUN043080	NAC
	MsUN045895	NAC
miR167	MsUN007721	AUX/IAA
miR169	MsUN004554	CCAAT
	MsUN014711	CCAAT
	MsUN030848	CCAAT
	MsUN044017	CCAAT
	MsUN096784	CCAAT
	MsUN102122	CCAAT
miR396	MsUN007041	GRF
	MsUN088040	GRF
	MsUN104762	C3HC4
	MsUN104763	C3HC4
	MsUN101411	MADS-box
miR2645	MsUN102295	bZIP
miR5205	MsUN046011	AP2-EREBP
miR5249	MsUN104998	bHLH
miR530	MsUN047511	NAC
NmiR0007	MsUN030863	GRAS
NmiR0028	MsUN045647	G2-like

The assembled alfalfa transcript sequences were submitted to the iTAK software for TF identification using default parameters; 28 TFs from 13 families were identified, and the results of hypergeometric test showed that TF genes were highly enriched among these targets.

**Figure 6:**
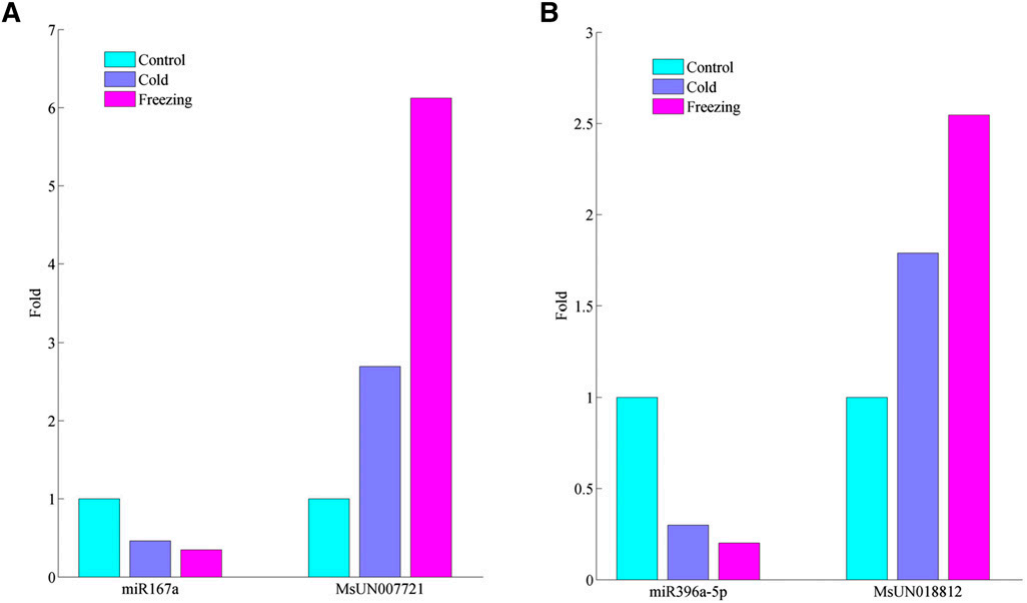
Differential expression of miRNAs and their target genes in response to cold and/or freezing stresses. (A) miRNA167a *vs.* MsUN007721; (B) miRNA396a-5p *vs.* MsUN018812. The miRNA expression abundances were evaluated by small RNA sequencing using the TPM method, while target genes were estimated by RNA-seq data using the FPKM method. Their expression levels were adjusted by comparing them with control samples, and relative fold change (FC) values were explored. FCs greater than 2 or less than 0.5 were identified as upregulated or downregulated, respectively.

Based on transcriptome sequencing, we evaluated the expression of the target genes under cold and freezing stresses. Among them, six target genes are regulated by four differentially expressed miRNAs (Table S7). Compared with control conditions, the expression of these four miRNAs was depressed under cold and freezing stress, and their targets (except MsUN037724, cleaved by miR396a-5p) were markedly upregulated under cold and freezing stresses, *i.e.*, MsUN007721, and MsUN018812 (Figure 6). In addition, correlation coefficients between miRNAs and their target genes were computed. Negative results (mean value close to –0.41) implied repressive regulation between an miRNA and its target gene. In particular, expression of the transcript MsUN007721 (an AUX/IAA TF), targeted by miRNA167a, was induced significantly under cold and freezing stress, which is consist with the observed repression of miRNA167a. These results suggested that miRNA167 plays an important role by regulating the functions of genes of the auxin signaling pathway (*i.e.*, MsUN007721) in alfalfa in response to cold and freezing stresses. A similar regulation function between miR167 and ARF TF has been confirmed as essential for soybean nodulation (Wang *et al.* 2015).

## Discussion

Plant growth and development are threatened by extreme environmental conditions worldwide. In response to stress, plants employ complex systems to adapt to environmental stress, undergoing physiological and biochemical changes in response to unfavorable conditions. MiRNAs are important regulators involved in various stress responses that have received increasing attention, especially with the development of high-throughput sequencing technology. Freezing stress is a common environmental stress of plants that affects plant growth, development, and survival, and particular limits perennial plants. Recently, miRNAs involved in freezing stress have been investigated in many plants, mainly in model plants such as *Arabidopsis* (Mallory and Vaucheret 2006; Sunkar and Zhu 2004), rice (Lv *et al.* 2010), wheat (Tang *et al.* 2012; Tang *et al.* 2011), and *M. truncatula* (Formey *et al.* 2014; Lelandais-Brière *et al.* 2009; Reynoso *et al.* 2013; Z. Zhou *et al.* 2008); however, few reports have been published about alfalfa, a perennial legume forage (Fan *et al.* 2014; Long *et al.* 2015). Here, we presented a comprehensive analysis of miRNAs, and their targets, in the freezing tolerant alfalfa cultivar Zhaodong, under cold and freezing stress, using high-throughput sequencing. The results revealed that miRNAs are indeed affected by freezing stress, implying their role in this process. In addition, targets of miRNAs, including TFs, enzymes, and nodulation genes, were identified by degradome sequencing. Taken together, these results provide novel insights into the regulatory mechanism of cold acclimation and freezing tolerance in alfalfa, mediated by miRNAs.

### Different expression patterns of miRNAs involving in freezing stress in alfalfa

Based on our sequencing results, 35 miRNAs were differentially regulated by cold and/or freezing stress in alfalfa. Notably, most miRNAs (29/35, 83%) were downregulated by cold and/or freezing stress, which is consist with previous reports in other plants (Chen *et al.* 2012; Long *et al.* 2015; Lv *et al.* 2010; Zhang *et al.* 2009; X. Zhou *et al.* 2008). Among these miRNAs, miRNAs responsive to cold stress were more conserved across plants, for instance, miR156, miR159, miR167, miR172, miR396, and miR398, implying their consistent function in cold stress. For example, the Cu/Zn superoxide dismutase-encoding gene, which is cleaved by miR398 in plants, acts as a scavenger of reactive oxygen species (ROS), playing an important role in plant abiotic stress (Dugas and Bartel 2008; Sunkar *et al.* 2006). Other miRNAs that were specifically regulated by freezing stress, and which were mainly Medicago-specific, included miR5231, miR5232, miR5234, miR5239, miR5287, and novel miRNAs (NmiR0019, NmiR0026, NmiR0028, NmiR0029, NmiR0043, NmiR0047, NmiR0053). The results suggested that they play important roles in the freezing tolerance of alfalfa. However, their functions have been reported rarely in Medicago or other plants, some were even identified in the present study for the first time. Thus, their regulatory functions need to be determined in depth, which would be valuable for breeding alfalfa with improved freezing tolerance. In addition, there were also some miRNAs that were upregulated by cold and/or freezing stress, such as miR160, miR166, miR171, miR2119, and miR5037, which might regulate genes negatively involved in the freezing tolerance of alfalfa.

### TF targeted by alfalfa miRNAs involved in freezing tolerance

With development of high-throughput sequencing technology, degradome sequencing has been used widely to identify targets of miRNAs in plants. In the present study, we identified 105 targets from cold stress, and freezing stress libraries. Based on the targets’ functional annotations, we found that conserved miRNAs were more likely to target TFs involved in regulating plant growth and stress response, which is consistent with previous reports in other plants (Aung *et al.* 2015; Fang *et al.* 2014; Tang *et al.* 2012; Wang *et al.* 2015) (see Table 4). Degradome sequencing identified TF members from families characterized as targets of miRNAs, including SBP, ARF, NAC and GRF. In *Arabidopsis* and rice, miR156 participates in plant growth and development by cleaving SBP TFs (Ling and Zhang 2012; Xing *et al.* 2010). Similarly, miR160 and miR167 target auxin response factors (ARF and AUX/IAA), involved in plant development process (Rubio-Somoza *et al.* 2009; Wang *et al.* 2015; Yang *et al.* 2006). In this study, eight SBP, ARF and AUX/IAA TFs were identified by high-throughput sequencing, which indicated that the conserved miRNAs might regulate alfalfa cold and freezing responses by controlling alfalfa developmental process, which would correlate with their functions in other model plants. Similarly, NAC TFs are plant-specific, with important roles in plant development and stress response processes. Two NAC TFs were identified as targets of miR164, which was slightly suppressed by cold stress, implying that NAC TFs were positively regulated during the cold response (Fang *et al.* 2014). In addition to targeting TFs, some genes involved in stress responses and metabolic processes were also cleaved by miRNAs in alfalfa. For instance, the pentatricopeptide repeat (PPR) protein is a negative regulator of abscisic acid (ABA) signaling, which has a positive on plant responses to abiotic stresses (Jiang *et al.* 2014; Sechet *et al.* 2015). In total, eight PPR genes were identified as targets of NmiR0018 and NmiR0041 (see Table S8), implying that these miRNAs function in cold and freezing stress by controlling the expression of PPR genes.

### Freezing response miRNAs and their targets involved in the nodulation process

As a legume crop, alfalfa is able to establish a symbiotic association with nitrogen-fixing bacteria, resulting in development of a novel plant organ, termed the root nodule (Soyano and Hayashi 2014). Within nodules, the symbiotic nitrogen fixation relationship between the plant and bacteria is sensitive to environmental stress, such as salt, drought, and cold stress (Kaur *et al.* 2014; Zhang *et al.* 2014). In particular, nodules of alfalfa are perennial, and are capable of continuous growth, helping alfalfa to undergo dormancy, and to survive hard winters. However, the molecular mechanisms of symbiotic nitrogen fixation (SNF) in response to cold stress have received relatively little attention. In Medicago, some TFs were characterized as being involved in nodulation, including GRAS (Cerri *et al.* 2012; Heckmann *et al.* 2006; Hirsch *et al.* 2009; Liu *et al.* 2011), AP2/ERF (Middleton *et al.* 2007), and CCAAT TFs (HAP2-1/NF-YA) (Battaglia *et al.* 2014; Laporte *et al.* 2014), etc. Recently, miRNAs were also demonstrated to participate in the nodulation process, such as miR169 and miR172, which target the NF-YA/HAP2 and AP2 TFs (Combier *et al.* 2006; Reynoso *et al.* 2013; Wang *et al.* 2014). In this study, we confirmed the relationship between miRNAs and mRNAs; for instance, GRAS, AP2/ERF, and CCAAT TFs were regulated by NmiR0007, miR5205, and miR169. Significantly, six CCAAT TFs were cleaved by miR169, as identified by degradome sequencing (see Table 2). The results confirmed the function of miR169 as regulating the expression of CCAAT TFs (NF-YA/HAP2), which implied that miR169 responded to cold stress via the SNF process (Lelandais-Brière *et al.* 2009; Zhang *et al.* 2014).

### Conclusions

In summary, we investigated miRNAs and their targets by high-throughput sequencing technology during the cold and freezing response of alfalfa. We identified the expression profiles of 197 miRNAs (173 known miRNAs, and 24 novel miRNAs); 35 miRNAs were identified as cold- and/or freezing-responsive miRNAs. Using degradome sequencing, 105 functional genes were found to be cleaved by these miRNAs; most of them were transcription factors involved in plant development and abiotic response processes, which implied important roles in alfalfa freezing tolerance. Some differentially expressed miRNAs and their targets were identified as participating in SNF, which indicated that SNF might aid freezing tolerance in alfalfa. These findings provided valuable information for exploring the molecular mechanisms of the cold and freezing response, and also represent a foundation for future application of miRNAs to improve freezing tolerance in alfalfa or related plants.

## Supplementary Material

Supporting Information
