# Supplementary material for: Genome-Wide Investigation of MicroRNAs and Their Targets in Response to Freezing Stress in Medicago sativa L., Based on High-Throughput Sequencing
Source: G3 (Bethesda). 2016 Jan 20;6(3):755–65. doi: 10.1534/g3.115.025981 (PMC4777136; doi:10.1534/g3.115.025981)
Supplement: Supporting Information [file supp_6_3_755__index.html]

Genome-Wide Investigation of MicroRNAs and Their Targets in Response to Freezing Stress in Medicago sativa L., Based on High-Throughput Sequencing — Supporting Information 

# Genome-Wide Investigation of MicroRNAs and Their Targets in Response to Freezing Stress in *Medicago sativa* L., Based on High-Throughput Sequencing

## Supporting Information for Shu *et al.*, 2016

**Files in this Data Supplement:**

- Figure S1 - Distribution of miRNA reads in three alfalfa libraries. (.pdf, 94 KB)
- Table S5 - Target genes of miRNAs identified by two degradome sequencing libraries. (.pdf, 79 KB)
- Table S6 - Results of GO terms enrichment analysis using topGO in alfalfa. (.pdf, 82 KB)
- Table S7 - The differentially expression of target genes in alfalfa response to cold and/or freezing stresses. (.pdf, 74 KB)
- Table S8 - Function annotation of miRNAs targets excluding TFs identified by degradome sequencing. (.pdf, 80 KB)
- Figure S2 - Length distribution of alfalfa assembly transcripts. (.pdf, 98 KB)
- Figure S3 - Sequence identity distribution of assembly transcripts in *Medicago sativa* compared to *Medicago truncatula*. (.pdf, 105 KB)
- Figure S4 - Distribution of sequencing reads in two degradome sequencing libraries. (.pdf, 90 KB)
- Figure S5 - Class distribution of miRNA targets identified by two degradome sequencing libraries. (.pdf, 93 KB)
- Table S1 - Primers sequences of the reverse transcription and quantitative real-time PCR experiments. (.pdf, 75 KB)
- Table S2 - Conservation of miRNA families across three libraries. (.pdf, 83 KB)
- Table S3 - Novel miRNAs identified in three small RNA sequencing libraries. (.pdf, 77 KB)
- Table S4 - The expressions of miRNA genes identified in three alfalfa libraries. (.pdf, 88 KB)
- File S1 - Degradome sequencing analysis results of cold library by CleaveLand4 software. (.txt, 280 KB)
- File S2 - Degradome sequencing analysis results of freezing library by CleaveLand4 software. (.txt, 224 KB)
