# Supplementary figures and images for: Genome-Wide Investigation of MicroRNAs and Their Targets in Response to Freezing Stress in Medicago sativa L., Based on High-Throughput Sequencing
Source: G3 (Bethesda). 2016 Jan 20;6(3):755–65. doi: 10.1534/g3.115.025981 (PMC4777136; doi:10.1534/g3.115.025981)

Supplements

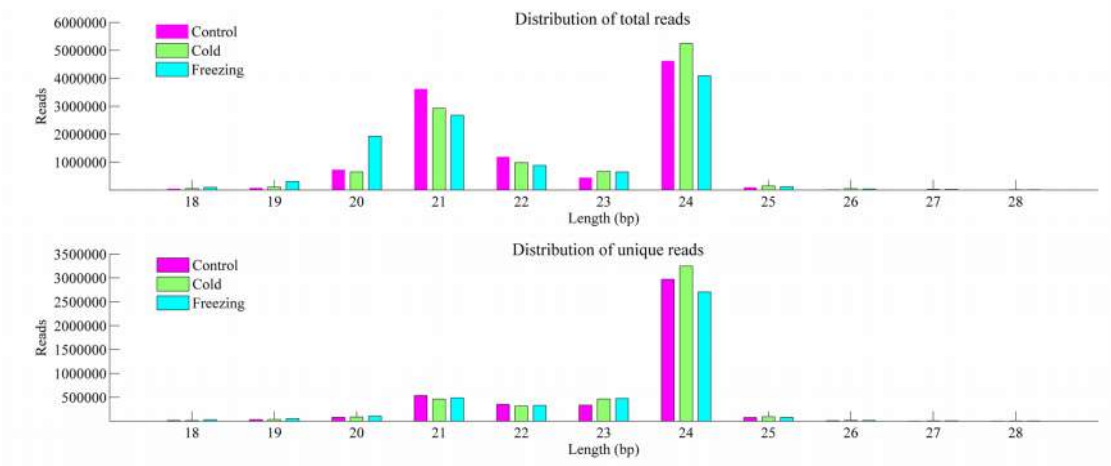

**Figure S1** Distribution of miRNA reads in three alfalfa libraries.

Supplement: Supporting Information [file supp_g3.115.025981_FigureS1.pdf]

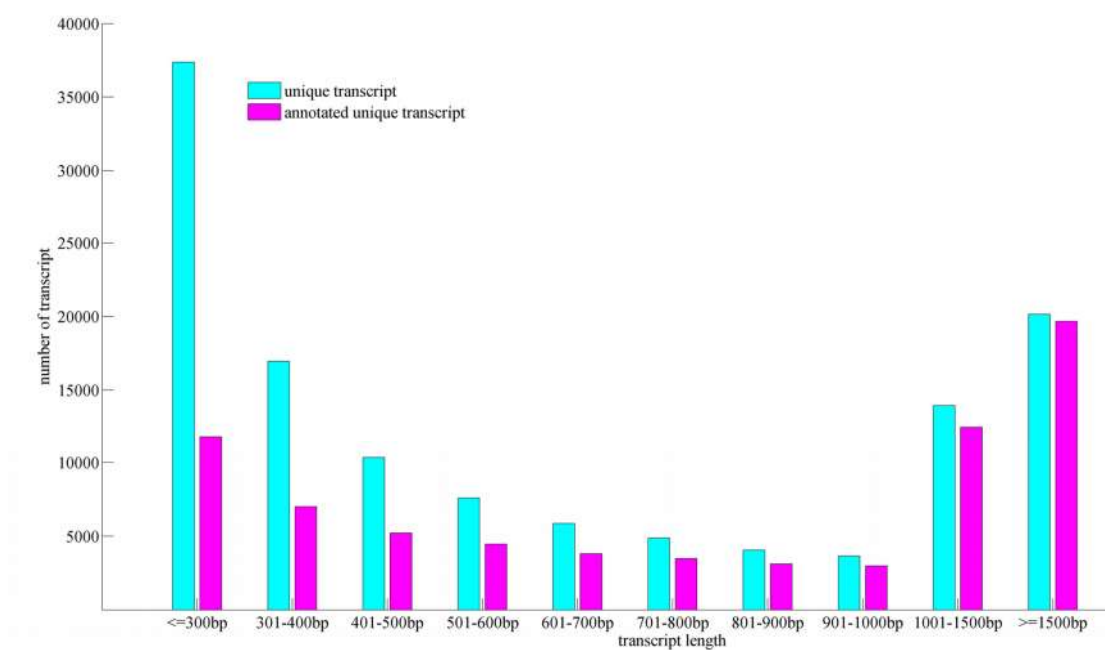

**Figure S2** Length distribution of alfalfa assembly transcripts.

Supplement: Supporting Information [file supp_g3.115.025981_FigureS2.pdf]

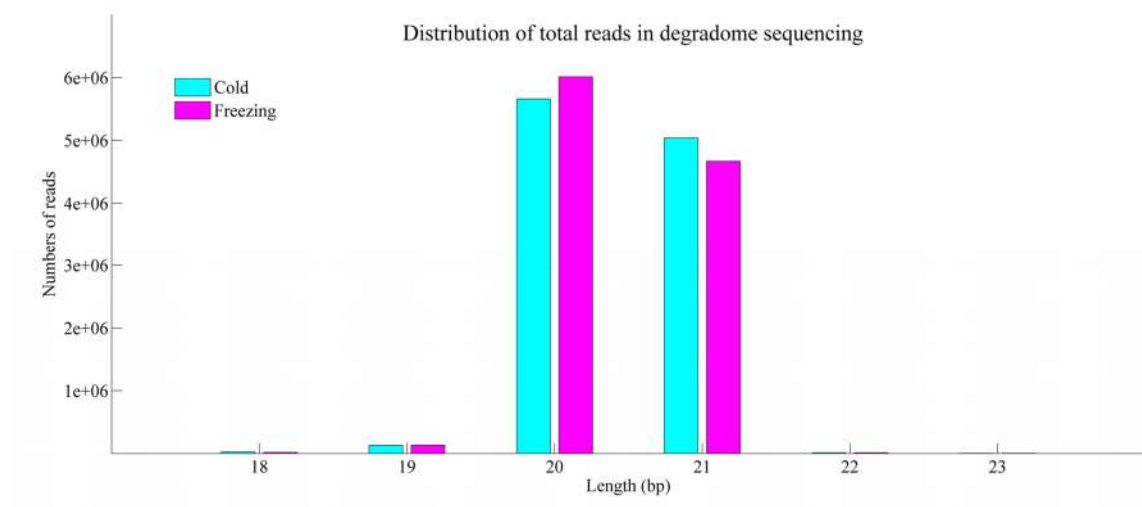

**Figure S4** Distribution of sequencing reads in two degradome sequencing libraries.

Supplement: Supporting Information [file supp_g3.115.025981_FigureS4.pdf]
