# Supplementary material for: Genome-Wide Investigation of MicroRNAs and Their Targets in Response to Freezing Stress in Medicago sativa L., Based on High-Throughput Sequencing
Source: G3 (Bethesda). 2016 Jan 20;6(3):755–65. doi: 10.1534/g3.115.025981 (PMC4777136; doi:10.1534/g3.115.025981)
Supplement: Supporting Information [file supp_g3.115.025981_TableS5.pdf]

Table S5 Target genes of miRNAs identified by two degradome sequencing libraries. The target genes were identified by two degradome libraries, cold and freezing samples. The "Yes" indicates function of the miRNA cleaving target genes was detected in corresponding degradome library, while "No" implies not detection.

miRNA	Target	Present in Cold	Present in Freezing
miR156	MsUN014236	Yes	Yes
	MsUN041240	Yes	No
	MsUN046666	Yes	No
	MsUN050762	No	Yes
	MsUN035352	Yes	No
	MsUN011072	No	Yes
	MsUN014236	Yes	No
	MsUN046666	Yes	No
	MsUN049469	Yes	Yes
miR160	MsUN086910	Yes	Yes
	MsUN020597	No	Yes
	MsUN020599	Yes	Yes
	MsUN041965	No	Yes
	MsUN097717	Yes	Yes
miR164	MsUN043080	Yes	Yes
	MsUN045895	No	Yes
	MsUN045895	Yes	No
miR167	MsUN007721	Yes	Yes
	MsUN010604	Yes	No
	MsUN012385	Yes	No
miR168	MsUN040125	No	Yes
	MsUN016316	No	Yes
	MsUN016316	Yes	No
miR169	MsUN004554	Yes	Yes
	MsUN014711	Yes	Yes
	MsUN030848	No	Yes
	MsUN044017	No	Yes
	MsUN096784	No	Yes
	MsUN102122	Yes	No
miR390	MsUN047287	Yes	Yes
miR396	MsUN007041	Yes	Yes
	MsUN018812	Yes	No
	MsUN037724	Yes	No
	MsUN038845	Yes	Yes
	MsUN047297	No	Yes
	MsUN050298	Yes	Yes
	MsUN088040	Yes	Yes

	MsUN104762	No	Yes
	MsUN104763	Yes	No
	MsUN009854	No	Yes
	MsUN009855	Yes	No
	MsUN101411	Yes	Yes
miR398	MsUN006428	No	Yes
	MsUN005180	Yes	No
	MsUN007372	Yes	Yes
	MsUN009260	No	Yes
	MsUN034743	Yes	Yes
	MsUN043960	No	Yes
miR1509	MsUN050005	Yes	No
	MsUN075066	Yes	No
	MsUN081284	Yes	Yes
	MsUN089687	Yes	No
	MsUN098062	Yes	Yes
	MsUN098063	Yes	Yes
miR2590	MsUN018931	No	Yes
	MsUN097071	Yes	No
miR2592	MsUN086833	Yes	Yes
	MsUN111458	No	Yes
miR2604	MsUN016528	No	Yes
miR2612	MsUN090041	No	Yes
miR2612	MsUN090042	Yes	No
miR2616	MsUN082240	No	Yes
	MsUN039546	Yes	Yes
miR2643	MsUN102436	Yes	No
	MsUN102457	No	Yes
miR2645	MsUN102295	No	Yes
miR5037	MsUN046365	Yes	No
miR5205	MsUN046011	Yes	No
	MsUN008813	Yes	Yes
	MsUN010070	Yes	No
	MsUN018393	No	Yes
miR5213	MsUN036655	Yes	No
	MsUN038569	Yes	No
	MsUN079376	Yes	Yes
	MsUN091135	Yes	No
miR5231	MsUN106315	Yes	No
miR5232	MsUN104761	No	Yes
	MsUN027490	Yes	No
miR5239	MsUN031868	Yes	No
	MsUN048390	Yes	Yes
miR5249	MsUN045450	Yes	Yes

	MsUN093768	No	Yes
	MsUN104998	No	Yes
miR5257	MsUN024706	No	Yes
miR5261	MsUN095228	Yes	No
miR5266	MsUN042197	Yes	No
miR5270	MsUN009940	No	Yes
miR5297	MsUN010427	Yes	No
miR530	MsUN047511	Yes	No
miR7696	MsUN085264	No	Yes
miR7701	MsUN045409	No	Yes
NmiR0006	MsUN040404	Yes	Yes
NmiR0007	MsUN030863	Yes	No
NmiR0018	MsUN005346	No	Yes
	MsUN038572	Yes	No
	MsUN105188	Yes	Yes
	MsUN115233	Yes	Yes
NmiR0022	MsUN020104	Yes	Yes
NmiR0026	MsUN102436	No	Yes
	MsUN102457	Yes	No
NmiR0027	MsUN014896	Yes	Yes
NmiR0028	MsUN045647	Yes	No
	MsUN093585	Yes	No
NmiR0029	MsUN093434	Yes	No
NmiR0041	MsUN019866	Yes	No
	MsUN023956	Yes	No
	MsUN032442	Yes	Yes
	MsUN046783	Yes	No
	MsUN094467	No	Yes
NmiR0053	MsUN089400	Yes	No
NmiR0063	MsUN113450	Yes	No
