# Supplementary material for: Genome-Wide Investigation of MicroRNAs and Their Targets in Response to Freezing Stress in Medicago sativa L., Based on High-Throughput Sequencing
Source: G3 (Bethesda). 2016 Jan 20;6(3):755–65. doi: 10.1534/g3.115.025981 (PMC4777136; doi:10.1534/g3.115.025981)
Supplement: Supporting Information [file supp_g3.115.025981_TableS6.pdf]

Table S6 Results of GO terms enrichment analysis using topGO in alfalfa. GO terms enrichment analysis of miRNA targeting genes in alfalfa was performed using topGO package of R, the p-value was calculated based Fisher test, and the significant level was set as 0.01.

Domain	GO ID	Term	Enrichment Genes	P-value
Biological Process	GO:0006351	transcription, DNA-templated	18	2.40E-11
	GO:0032774	RNA biosynthetic process	18	2.70E-11
	GO:0006355	regulation of transcription, DNA-templat...	17	2.80E-11
	GO:2001141	regulation of RNA biosynthetic process	17	2.80E-11
	GO:0051252	regulation of RNA metabolic process	17	2.90E-11
	GO:0010556	regulation of macromolecule biosynthetic...	17	3.60E-11
	GO:2000112	regulation of cellular macromolecule bio...	17	3.60E-11
	GO:0031326	regulation of cellular biosynthetic proc...	17	3.80E-11
	GO:0009889	regulation of biosynthetic process	17	3.90E-11
	GO:0019219	regulation of nucleobase-containing comp...	17	6.90E-11
	GO:0051171	regulation of nitrogen compound metaboli...	17	7.30E-11
	GO:0010468	regulation of gene expression	17	9.40E-11
	GO:0080090	regulation of primary metabolic process	17	9.60E-11
	GO:0034654	nucleobase-containing compound biosynthe...	18	1.00E-10
	GO:0031323	regulation of cellular metabolic process	17	1.20E-10
	GO:0060255	regulation of macromolecule metabolic pr...	17	1.30E-10
	GO:0018130	heterocycle biosynthetic process	18	3.60E-10
	GO:0044271	cellular nitrogen compound biosynthetic ...	18	3.70E-10
	GO:0019438	aromatic compound biosynthetic process	18	4.00E-10
	GO:1901362	organic cyclic compound biosynthetic pro...	18	8.40E-10
	GO:0019222	regulation of metabolic process	17	1.10E-09
	GO:0050794	regulation of cellular process	23	1.40E-09
	GO:0065007	biological regulation	24	3.20E-09
	GO:0016070	RNA metabolic process	18	6.00E-09
	GO:0050789	regulation of biological process	23	7.40E-09
	GO:0034645	cellular macromolecule biosynthetic proc...	20	1.80E-08
	GO:0009059	macromolecule biosynthetic process	20	1.90E-08
	GO:0090304	nucleic acid metabolic process	18	6.80E-07
	GO:0044249	cellular biosynthetic process	20	7.10E-07
	GO:1901576	organic substance biosynthetic process	20	9.20E-07
	GO:0010467	gene expression	20	1.00E-06
	GO:0009058	biosynthetic process	20	3.20E-06
	GO:0006139	nucleobase-containing compound metabolic...	18	4.10E-06
	GO:0034641	cellular nitrogen compound metabolic pro...	18	8.90E-06
	GO:0046483	heterocycle metabolic process	18	9.00E-06
	GO:0006725	cellular aromatic compound metabolic pro...	18	1.00E-05

	GO:1901360	organic cyclic compound metabolic proces...	18	1.40E-05
	GO:0006807	nitrogen compound metabolic process	18	3.30E-05
	GO:0015979	photosynthesis	4	5.70E-05
	GO:0009725	response to hormone	5	0.00058
	GO:0009719	response to endogenous stimulus	5	0.00067
	GO:0044237	cellular metabolic process	27	0.00076
	GO:0010033	response to organic substance	5	0.00181
	GO:0010249	auxin conjugate metabolic process	1	0.00205
	GO:0042221	response to chemical	6	0.00233
	GO:0044260	cellular macromolecule metabolic process	22	0.00352
	GO:0009987	cellular process	32	0.0042
	GO:0050896	response to stimulus	14	0.00421
	GO:0043170	macromolecule metabolic process	23	0.00638
Molecular Function	GO:0003677	DNA binding	17	3.90E-07
	GO:1901363	heterocyclic compound binding	40	6.70E-05
	GO:0097159	organic cyclic compound binding	40	6.90E-05
	GO:0001071	nucleic acid binding transcription facto...	9	0.00024
	GO:0003700	sequence-specific DNA binding transcript...	9	0.00024
	GO:0043531	ADP binding	14	0.00072
	GO:0003676	nucleic acid binding	18	0.0008
	GO:0005488	binding	55	0.00108
Cellular Component	GO:0005634	nucleus	17	2.10E-08
	GO:0009522	photosystem I	3	1.50E-05
	GO:0005622	intracellular	26	1.70E-05
	GO:0044424	intracellular part	25	2.10E-05
	GO:0043227	membrane-bounded organelle	21	5.40E-05
	GO:0043231	intracellular membrane-bounded organelle	21	5.40E-05
	GO:0005623	cell	28	7.40E-05
	GO:0044464	cell part	28	7.40E-05
	GO:0009538	photosystem I reaction center	2	8.10E-05
	GO:0043226	organelle	21	0.00048
	GO:0043229	intracellular organelle	21	0.00048
	GO:0009521	photosystem	3	0.00059
	GO:0034357	photosynthetic membrane	4	0.00154
	GO:0044436	thylakoid part	4	0.00226
	GO:0009579	thylakoid	4	0.00301
