# Supplementary material for: Genome-Wide Investigation of MicroRNAs and Their Targets in Response to Freezing Stress in Medicago sativa L., Based on High-Throughput Sequencing
Source: G3 (Bethesda). 2016 Jan 20;6(3):755–65. doi: 10.1534/g3.115.025981 (PMC4777136; doi:10.1534/g3.115.025981)
Supplement: Supporting Information [file supp_g3.115.025981_TableS7.pdf]

Table S7 The differentially expression of target genes in alfalfa response to cold and/or freezing stresses. Expressions of target genes (FPKM method, fragments per kilobase of exon per million fragments mapped) were calculated by TopHat and Cufflinks. Differential expression of target genes were identified by package edger, with |log2(fold change)| ≥1 and p-value ≤0.01. The correlations (Person correlation coefficients) between expression of miRNA and their target genes were analysis using R (cor commond).

miRNA	Target	Expression			Correlation
		Control	Cold	Freezing	
miR167a	MsUN007721	7.54	20.31	46.15	-0.85
miR396a-5p	MsUN018812	2.71	4.84	6.90	-0.92
miR396a-5p	MsUN037724	7.21	2.55	5.90	0.64
miR396a-5p	MsUN038845	3.62	0.68	12.28	-0.38
miR5239	MsUN031868	3.14	0.99	7.41	-0.50
NmiR0028	MsUN045647	25.91	128.50	37.47	-0.46
