# Supplementary material for: Genome-Wide Investigation of MicroRNAs and Their Targets in Response to Freezing Stress in Medicago sativa L., Based on High-Throughput Sequencing
Source: G3 (Bethesda). 2016 Jan 20;6(3):755–65. doi: 10.1534/g3.115.025981 (PMC4777136; doi:10.1534/g3.115.025981)
Supplement: Supporting Information [file supp_g3.115.025981_TableS8.pdf]

Table S8 Function annotation of miRNAs targets excluding TFs identified by degradome sequencing. The target genes were firstly scanned TF genes by iTAK software, and remains were annotated by BLASTX against combination databases of Arabidopsis, rice, soybean, and Medicago truncatula proteins. The annotation information of hits with e-value lower than 1E-5 were retrieved.

miRNA	Target	Annotation information
miR160	MsUN020597	alpha/beta-Hydrolases superfamily protein
	MsUN020599	alpha/beta-Hydrolases superfamily protein
miR167	MsUN012385	Transducin/WD40 repeat-like superfamily protein
miR168	MsUN040125	dehydrin family protein
	MsUN016316	Galactose oxidase/kelch repeat superfamily protein
miR396	MsUN050298	F-box/RNI-like superfamily protein
miR398	MsUN006428	Terpenoid cyclases/Protein prenyltransferases superfamily protein
miR1509	MsUN050005	Carbohydrate-binding X8 domain superfamily protein
	MsUN081284	Subtilase family protein
miR2592	MsUN097071	reduced male fertility
	MsUN086833	Protein kinase family protein
	MsUN111458	F-box and associated interaction domains-containing protein
miR2612	MsUN090041	cofactor of nitrate reductase and xanthine dehydrogenase 2
	MsUN090042	cofactor of nitrate reductase and xanthine dehydrogenase 2
miR2616	MsUN082240	DNAJ heat shock N-terminal domain-containing protein
miR2643	MsUN039546	Protein kinase superfamily protein
	MsUN102436	Small nuclear ribonucleoprotein family protein
	MsUN102457	Small nuclear ribonucleoprotein family protein
miR5037	MsUN046365	production of anthocyanin pigment 1
miR5213	MsUN008813	aspartate aminotransferase 4
	MsUN010070	histidine triad nucleotide-binding 4
	MsUN018393	ARM repeat superfamily protein
	MsUN036655	unknown protein
	MsUN038569	Ankyrin repeat family protein
	MsUN079376	succinate dehydrogenase subunit 4
	MsUN091135	unknown protein
miR5231	MsUN106315	PDI-like 5-1
miR5239	MsUN031868	Putative serine esterase family protein
miR5239	MsUN048390	Putative serine esterase family protein
miR5249	MsUN045450	ubiquitin-conjugating enzyme 13
miR5257	MsUN024706	galacturonosyltransferase 10
miR5261	MsUN095228	N-acetyl-l-glutamate kinase
miR5266	MsUN042197	KAR-UP F-box 1
miR5270	MsUN009940	germin-like protein subfamily 2 member 2 precursor
miR7701	MsUN045409	Cytochrome bd ubiquinol oxidase, 14kDa subunit
NmiR0006	MsUN040404	inositol-pentakisphosphate 2-kinase 1
NmiR0018	MsUN005346	Pentatricopeptide repeat (PPR) superfamily protein

	MsUN038572	Pentatricopeptide repeat (PPR) superfamily protein
	MsUN105188	Pentatricopeptide repeat (PPR) superfamily protein
	MsUN115233	Pentatricopeptide repeat (PPR) superfamily protein
NmiR0026	MsUN102436	Small nuclear ribonucleoprotein family protein
	MsUN102457	Small nuclear ribonucleoprotein family protein
NmiR0027	MsUN014896	Tetratricopeptide repeat (TPR)-like superfamily protein
NmiR0029	MsUN093434	alpha/beta-Hydrolases superfamily protein
	MsUN019866	Pentatricopeptide repeat (PPR) superfamily protein
	MsUN023956	Pentatricopeptide repeat (PPR) superfamily protein
NmiR0041	MsUN032442	Pentatricopeptide repeat (PPR) superfamily protein
	MsUN046783	Pentatricopeptide repeat (PPR) superfamily protein
	MsUN094467	MAP kinase 9
NmiR0053	MsUN089400	F-box and associated interaction domains-containing protein
NmiR0063	MsUN113450	BSD domain-containing protein
