# Supplementary material for: Genome-Wide Investigation of MicroRNAs and Their Targets in Response to Freezing Stress in Medicago sativa L., Based on High-Throughput Sequencing
Source: G3 (Bethesda). 2016 Jan 20;6(3):755–65. doi: 10.1534/g3.115.025981 (PMC4777136; doi:10.1534/g3.115.025981)
Supplement: Supporting Information [file supp_g3.115.025981_TableS1.pdf]

Table S1 Primers sequences of the reverse transcription and quantitative real-time PCR experiments

miRNA	RT primer	Quantitative real-time PCR primer	
		Forward Primer	Reversed Primer
miR156i-5p	GTCGTATCCAGTGCGTGTCGTGGAGTCGGCAATTGCACTGGATACGACGTGCTC	GCGCGTGACAGAAGAGAGTG	TATCCAGTGCGTGTCGTGG
miR160e	GTCGTATCCAGTGCGTGTCGTGGAGTCGGCAATTGCACTGGATACGACTGGCAT	GTGCCTGGCTCCCTGTATG	TATCCAGTGCGTGTCGTGG
miR166f	GTCGTATCCAGTGCGTGTCGTGGAGTCGGCAATTGCACTGGATACGACGAGGAA	CGTCGGACCAGGCTTCAT	TATCCAGTGCGTGTCGTGG
miR167a	GTCGTATCCAGTGCGTGTCGTGGAGTCGGCAATTGCACTGGATACGACTAGATC	CGTGAAGCTGCCAGCATG	TATCCAGTGCGTGTCGTGG
miR172c-3p	GTCGTATCCAGTGCGTGTCGTGGAGTCGGCAATTGCACTGGATACGACATGCAG	CGCGAGAATCTTGATGATG	GTCGTGGAGTCGGCAATT
miR398a-5p	GTCGTATCCAGTGCGTGTCGTGGAGTCGGCAATTGCACTGGATACGACCTTGTG	CGCGGGAGTGACACTGAG	TATCCAGTGCGTGTCGTGG
miR396a-5p	GTCGTATCCAGTGCGTGTCGTGGAGTCGGCAATTGCACTGGATACGACAAGTTC	GCGTTCCACAGCTTTCTTG	TATCCAGTGCGTGTCGTGG
miR5037c	GTCGTATCCAGTGCGTGTCGTGGAGTCGGCAATTGCACTGGATACGACCCGTGG	CGCAACCCTCAAAGGCTTC	TATCCAGTGCGTGTCGTGG
miR5231	GTCGTATCCAGTGCGTGTCGTGGAGTCGGCAATTGCACTGGATACGACTGAGCT	GCGGCGTTATGCAAGTAGAT	TATCCAGTGCGTGTCGTGG
NmiR0018	GTCGTATCCAGTGCGTGTCGTGGAGTCGGCAATTGCACTGGATACGACTTAGAT	CGCGTTTCGTTCCATACATC	TATCCAGTGCGTGTCGTGG
NmiR0026	GTCGTATCCAGTGCGTGTCGTGGAGTCGGCAATTGCACTGGATACGACTCTCTA	GGCGTTTGGGATCAGAAAT	TATCCAGTGCGTGTCGTGG
NmiR0051	GTCGTATCCAGTGCGTGTCGTGGAGTCGGCAATTGCACTGGATACGACTGGCCT	GCGCGTGAATTTAGAGCTAG	GTCGTGGAGTCGGCAATT
U6	CCAAAACTTCACTTATG	TCGGGGACATCCGATAAAAT	TTTGTGCGTGTCATCCTTGC
