# Supplementary material for: Genome-Wide Investigation of MicroRNAs and Their Targets in Response to Freezing Stress in Medicago sativa L., Based on High-Throughput Sequencing
Source: G3 (Bethesda). 2016 Jan 20;6(3):755–65. doi: 10.1534/g3.115.025981 (PMC4777136; doi:10.1534/g3.115.025981)
Supplement: Supporting Information [file supp_g3.115.025981_TableS2.pdf]

Table S2 Conservation of miRNA families across three libraries

miRNA family	miRNA genes	Control	Cold	Freezing
miR156	mtr-miR156a, mtr-miR156b-3p, mtr-miR156c-3p, mtr-miR156d-3p, mtr-miR156g-3p, mtr-miR156g-5p, mtr-miR156h-5p, mtr-miR156i-3p, mtr-miR156i-5p, mtr-miR156j	9	9	10
miR159	mtr-miR159a, mtr-miR159b	1	1	2
miR160	mtr-miR160c, mtr-miR160e	2	2	2
miR164	mtr-miR164c, mtr-miR164d	2	2	2
miR166	mtr-miR166e-5p, mtr-miR166f, mtr-miR166g-3p, mtr-miR166g-5p	4	4	4
miR167	mtr-miR167a, mtr-miR167b-5p	2	2	2
miR168	mtr-miR168c-3p, mtr-miR168c-5p	2	2	2
miR169	mtr-miR169e-5p, mtr-miR169f, mtr-miR169l-5p	3	3	3
miR171	mtr-miR171a, mtr-miR171b, mtr-miR171c, mtr-miR171d, mtr-miR171e-3p, mtr-miR171f	6	6	6
miR172	mtr-miR172a, mtr-miR172c-3p, mtr-miR172c-5p, mtr-miR172d-3p, mtr-miR172d-5p	4	4	4
miR390	mtr-miR390	1	1	1
miR393	mtr-miR393b-5p	1	1	1
miR395	mtr-miR395o	1	1	1
miR396	mtr-miR396a-5p, mtr-miR396b-3p, mtr-miR396b-5p	3	3	3
miR397	mtr-miR397-5p	1	1	1
miR398	mtr-miR398a-3p, mtr-miR398a-5p, mtr-miR398c	3	3	3
miR399	mtr-miR399i, mtr-miR399t-3p	1	0	1
miR408	mtr-miR408-3p, mtr-miR408-5p	2	2	2
miR1507	mtr-miR1507-3p	1	1	1
miR1509	mtr-miR1509b	1	1	1
miR1510	mtr-miR1510a-3p, mtr-miR1510a-5p, mtr-miR1510b-3p, mtr-miR1510b-5p	4	4	4
miR2089	mtr-miR2089-5p	0	0	1
miR2118	mtr-miR2118	1	1	1
miR2119	mtr-miR2119	1	1	1
miR2199	mtr-miR2199	1	1	1
miR2585	mtr-miR2585d	1	1	1
miR2586	mtr-miR2586a	1	1	1
miR2587	mtr-miR2587g	1	1	1
miR2590	mtr-miR2590j	1	1	1
miR2591	mtr-miR2591	1	1	1
miR2592	mtr-miR2592a-3p, mtr-miR2592am, mtr-miR2592bl-5p, mtr-miR2592bn-5p,	7	7	7

	mtr-miR2592bo-5p, mtr-miR2592br-3p, mtr-miR2592s-5p			
miR2593	mtr-miR2593d	1	0	1
miR2597	mtr-miR2597	1	1	1
miR2598	mtr-miR2598	1	1	1
miR2601	mtr-miR2601	1	1	1
miR2603	mtr-miR2603	1	1	1
miR2604	mtr-miR2604	1	1	1
miR2606	mtr-miR2606b, mtr-miR2606c	2	1	2
miR2608	mtr-miR2608	1	1	1
miR2612	mtr-miR2612	0	1	0
miR2614	mtr-miR2614	1	1	1
miR2616	mtr-miR2616	1	1	1
miR2625	mtr-miR2625	1	1	1
miR2627	mtr-miR2627	0	0	1
miR2629	mtr-miR2629g	1	1	1
miR2630	mtr-miR2630v	1	1	1
miR2638	mtr-miR2638b	1	0	1
miR2645	mtr-miR2645	1	1	1
miR2651	mtr-miR2651	1	1	1
miR2652	mtr-miR2652m	0	0	1
miR2655	mtr-miR2655o	1	1	1
miR2666	mtr-miR2666	1	1	1
miR2670	mtr-miR2670d	1	1	1
miR2673	mtr-miR2673b	1	0	1
miR2678	mtr-miR2678	1	1	0
miR5037	mtr-miR5037c	1	1	1
miR5205	mtr-miR5205a, mtr-miR5205b, mtr-miR5205d	3	3	3
miR5207	mtr-miR5207	1	1	1
miR5208	mtr-miR5208c, mtr-miR5208d	2	2	2
miR5213	mtr-miR5213-3p, mtr-miR5213-5p	1	2	1
miR5222	mtr-miR5222	1	1	1
miR5228	mtr-miR5228	1	1	1
miR5230	mtr-miR5230	1	1	1
miR5231	mtr-miR5231	1	1	1
miR5232	mtr-miR5232	1	1	1
miR5234	mtr-miR5234	1	1	1
miR5237	mtr-miR5237	1	1	1
miR5238	mtr-miR5238	1	1	1
miR5239	mtr-miR5239	1	1	1
miR5241	mtr-miR5241c	1	1	1
miR5242	mtr-miR5242	1	1	1
miR5243	mtr-miR5243	1	1	0
miR5244	mtr-miR5244	1	1	1

miR5245	mtr-miR5245	1	1	1
miR5248	mtr-miR5248	1	1	1
miR5249	mtr-miR5249	1	1	1
miR5254	mtr-miR5254	1	0	0
miR5255	mtr-miR5255	1	1	1
miR5256	mtr-miR5256	1	1	1
miR5257	mtr-miR5257	1	1	1
miR5261	mtr-miR5261	1	1	1
miR5266	mtr-miR5266	1	1	1
miR5267	mtr-miR5267n, mtr-miR5267o	2	2	2
miR5269	mtr-miR5269a, mtr-miR5269b	1	1	2
miR5272	mtr-miR5272e, mtr-miR5272f	2	2	2
miR5273	mtr-miR5273	1	1	1
miR5277	mtr-miR5277	0	1	1
miR5279	mtr-miR5279	1	1	1
miR5281	mtr-miR5281a, mtr-miR5281f	2	2	2
miR5282	mtr-miR5282	1	1	1
miR5283	mtr-miR5283	1	1	1
miR5284	mtr-miR5284b, mtr-miR5284g, mtr-miR5284h	2	3	2
miR5286	mtr-miR5286a, mtr-miR5286b	2	2	2
miR5287	mtr-miR5287a, mtr-miR5287b	2	2	2
miR5290	mtr-miR5290	1	1	1
miR5291	mtr-miR5291c	1	0	0
miR5292	mtr-miR5292a	1	1	1
miR5294	mtr-miR5294c	1	1	1
miR5295	mtr-miR5295d	1	1	1
miR5297	mtr-miR5297	1	1	1
miR5298	mtr-miR5298c, mtr-miR5298d	2	2	2
miR5299	mtr-miR5299	1	1	1
miR530	mtr-miR530	1	1	1
miR5561	mtr-miR5561-3p	1	1	0
miR5741	mtr-miR5741e	1	1	0
miR5743	mtr-miR5743b	1	0	1
miR5744	mtr-miR5744	1	1	1
miR5745	mtr-miR5745a, mtr-miR5745b	2	2	2
miR5756	mtr-miR5756	1	0	0
miR7696	mtr-miR7696c-5p, mtr-miR7696d-3p, mtr-miR7696d-5p	2	3	1
miR7699	mtr-miR7699-5p	1	1	1
miR7701	mtr-miR7701-3p, mtr-miR7701-5p	2	2	2
