# Supplementary material for: Genome-Wide Investigation of MicroRNAs and Their Targets in Response to Freezing Stress in Medicago sativa L., Based on High-Throughput Sequencing
Source: G3 (Bethesda). 2016 Jan 20;6(3):755–65. doi: 10.1534/g3.115.025981 (PMC4777136; doi:10.1534/g3.115.025981)
Supplement: Supporting Information [file supp_g3.115.025981_TableS3.pdf]

Table S3 Novel miRNAs identified in three small RNA sequencing libraries

miRNA	Hairpin sequence	Mature sequence
NmiR0005	AUAAGGCGAAGUUCGUCACUGGAUGCAGCGGUUCAUCGAUCUGUUCCUGAAUUUUGUUUGUCUCGCAAAACAAACAUGAAUCGGUCGAUAAACCUCUGCAUCCAGCGCUCACUUUUCCCUCUCUU	UCGAUAAACCUCUGCAUCCAG
NmiR0006	GCAAUUAACUUUCGUCGUCAUGGUGGUAACAUGAAUGGUCAGAUUUUGCUCAUAUUGCAAAGAUCUGAACAAUCUUGUUAUCAUCUGGUGAAGAAAGUUUUAUGCU	UUCGUCGUCAUGGUGGUAACAUG
NmiR0008	CAUGCAUACAUAUGUGUAUGUGCCUGGCUCCCUGUAUGCCAUAUACGAAGCUGGUUGAAAAAUCAACGACCUUUGUAGAUGGCGUAUGAGGAGCCAAGCAUAUUCCAUGUCCAUACAUGGAG	GCGUAUGAGGAGCCAAGCAUA
NmiR0009	GUUUGAGGAACUUUUCAUGUUCCCUCAAAGGCUUCCAGUAUUCGGUCAUCUAAUUACUUGAAUGCUGGAAGGUUUUGUAGGAACUUGAUAGCUUCUUCUAUGUG	GCUGGAAGGUUUUGUAGGAAC
NmiR0015	UUCUGCAGAAGUAGACAGGAGUGGUGUUGUUUCUGCUCAUCUUAUUAAAUAAUGAACGAAUGACAUUUGUUAAAUUAUAAGACGAGCCGAAUCAAUAUCACUCCGGUACACCUCACUGCAUGC	CGAGCCGAAUCAAUAUCACUC
NmiR0018	GAAUUAAUGCAAUGUUGCUUAGAGAUAUAUGGAAUGAAAGAUGUGAAUUUCUUUUUGGAUUCUAUCCAUUCAAUUUUCUCUCUUUCGUUCCAUACAUCAUCUAACCAACAAACUGUUAAUCUGG	UUUCGUUCCAUACAUCAUCUAA
NmiR0019	UUCAAGUCCUGGUCAUGCUUUUCCACAGCUUUCUUGAACUUCUUUCGUAUCUUAAAUCUGUUUUCAAGAUUAAAAGCCCUAGAAGCUCAAGAAAGCUGUGGGAGAAUAUGGCAAUUCAGGCUUCA	GCUCAAGAAAGCUGUGGGAGA
NmiR0022	CACCCAUCCAUGACCUCAUCUUUUCCAAUUCUAAGUCUAUCUGACUAUCCCUAUGAAUUCUGAGAUAGAUUUAGAAUUGGAAAAGCUAAGUUCCAUGGGAUGGAUU	UUUUCCAAUUCUAAGUCUAUC
NmiR0026	CUCUAAUCUCUGAUCCCAAAUACGCUAAAUCCAUAAAAAGAUCAAGUGAUUUUGGGAUCAGAAAUUAGAGAUAUCAAGUACUUGCAACCAG	UUUGGGAUCAGAAAUUAGAGA
NmiR0027	AGUGUUGCUUACACAUUUUUUUUGGCAUUCUGUCCACCUCCUCUAUCCUUAUUUUCCUUGAUCGAUCUCAUCACUAAUUUGCGACCGGAGGUGGACAAACUGUCAACAGAGUUGUGUUGGCUUCUCUUU	UUUGGCAUUCUGUCCACCUCC
NmiR0028	GGAAACAGAACACUGCUCUUUUCAUUUCUAAAAUAGGCAUUGCAAUUCAAAUUCCAUGGAUCCAUUCAGCAAUGCCUAUUUUAGAAUGAAAACAACAUUUCACUGUUUCCAU	UUCAUUUCUAAAAUAGGCAUUG
NmiR0029	GCUUCAGACAUAAGUAUGCUUCAAGUUCUCAUAAUUCAAAAAGACAAGCUACAUUUGAAAAACUUACAUAAACUUUUCGUUUUGAAUUAUGAGAACUUGAAGAAGUCUAAAGCUAGCUCAGA	UUGAAUUAUGAGAACUUGAAG
NmiR0033	AUGGUUUUGCUACAAAUUCACCAUCUUUCUAGUUCCGCAUUUAUGUCUUGCUGCUUGAAUUUAAGGGAAAUCAAGCAUGGACAUUAAUGCUGAGGUAGAAAGAUGGUCCGAGACAAUUUGAGCGAGAA	UGCUGAGGUAGAAAGAUGGUC
NmiR0037	GUAGAUUUGAGCCAAAGAUGACUUGCCGGCAUGCUGCCUGAAAAGCAAGAAGGGUGUUGUUUUGCUGGCAGUUGUCAUUGGUUCAUGUUUGCUCUUUUCACUCUCAU	UGAGCCAAAGAUGACUUGCCGG
NmiR0041	UCCUUAAUGCGGUGUUGGGUAGAGUCGUAUGGAGUGAAAGAUGGGAAUUUCUUGUUGGAUUCUAUUCAUUCAAUUUUCCCUCUUUCAUUCCAUAUGUCAUCUAGCUAGCAUAUAUAGCGUUAAC	UUUCAUUCCAUAUGUCAUCUAG
NmiR0043	ACAAAUAGGACCUAGGAACUUCAAGUGCUAGUGCAAAUCUCCCACAAACGGGGAGAUUUGUUGAGGGGGAGAUUUCCACUAGCACUUGAAGUUCCUGGGUCGAUUAAUUAAUCGA	UCCACUAGCACUUGAAGUUCC
NmiR0047	UUGUACGAUGGAUGCAAUUUUGAACACUGGCUUGUUGUACAGUUAUUAUUACCUUCCCAUAACAUUAGCUAAAUAAUCACUGUACAACAAGCCAGUGUUCAAAAUUGCAUCCAUCGUACAAAC	UGAACACUGGCUUGUUGUACA
NmiR0049	CAUCAUGCACCAUACCAAGUUGAAGCUGCCAGCAUGAUCUUAACUGACCUCCUUUGUAGGUGAAAGAUCAGAUCAUGGUGGCAGUUUCACCUAGUUGUUGGCUGCAUGU	UGAAGCUGCCAGCAUGAUCUUAA
NmiR0051	CAUCUUCAAAAAGUCUUUUCUGAAUUUAGAGCUAGAGGCCAUCUUGGAGGAGAAAGUUGUCUACCUAAUUAGAUUUAGAGGCAAAACCUAUGCCAUAUUUGAUAUCAAGAUUGCCUCUAGUUCUAAAAUCACAAAAGGCUUAUUGAAGAAU	UGAAUUUAGAGCUAGAGGCCA
NmiR0053	CACAGUCGUUGUUUGCAGAUGUAGCAUCAUCAAGAUUCAUAUGUGAAUGAUGCAGUGGAACUGCUAAUACUUCCAUUGAUCUUUUGAAAUGAGAAUCUUGAUGAUGCUGCAUCAGCCAUAUACGACUUUAUA	GUAGCAUCAUCAAGAUUCAUA
NmiR0057	AAUUCUUGUUAGGCUGGUUUAGAUGAAGGUAUUAGGUGUCAAGGAAUGGACCUCAUUUCGUUGGCACUUGAAUACCAUCAUCUAAAUCAAAUCUGACAAAAAUUU	AGGCUGGUUUAGAUGAAGGUA
NmiR0058	GUCGAGUUUCUCAACUCGCAUUCCAAGUUGGAGAUUGUGGACAUGUUAAGAAAACACACAUACAAUGACGAGCUGGAACAGAGCAAGCGCAACCGUGGCU	UUCCAAGUUGGAGAUUGUGGA
NmiR0062	AACAGUCAGUGUUUGCUAGUGGAGCAUCAUCAAGAUUCACAAGCUUUAAAGGGCUUCAAAUCAAUUAGCCCUUCUUUAUGUUAUGAGAAUCUUGAUGAUGCUGCAGCAGCAAUUGAUGACUAAUACUACC	GGAGCAUCAUCAAGAUUCACA
NmiR0063	UGCACAGCUUGGAUGUACAUAUAAUUUAUAUCAUCUUGUGGUCAACGAGAAACAAUGAAAUUAUGUGUCCAUCUUCGUCUGGGUUUAUGUCCACUUUUUACUG	UAUGUGUCCAUCUUCGUCUGGG
