# Supplementary material for: Genome-Wide Investigation of MicroRNAs and Their Targets in Response to Freezing Stress in Medicago sativa L., Based on High-Throughput Sequencing
Source: G3 (Bethesda). 2016 Jan 20;6(3):755–65. doi: 10.1534/g3.115.025981 (PMC4777136; doi:10.1534/g3.115.025981)
Supplement: Supporting Information [file supp_g3.115.025981_TableS4.pdf]

Table S4 The expressions of miRNA genes identified in three alfalfa libraries. The miRNA abundance was evaluated and normalized using TPM (tags per million reads) method based on blast mapping results, and the TPM values were calculated as follows: TPM=number of mapped miRNA reads*10^6/number of clean sample reads. The normalized expression was adjusted to 0.01 when miRNA expression (TPM) was zero.

miRNA	Control	Cold	Freezing
miR1507-3p	2.96	1.11	2.6
miR1509b	2.77	1.11	2.6
miR1510a-3p	339.18	291.52	190.52
miR1510a-5p	60.98	61.76	90.25
miR1510b-3p	39.18	37.02	44.61
miR1510b-5p	0.18	0.28	0.46
miR156a	1349.53	1252.65	1316.1
miR156b-3p	0.74	2.77	2.6
miR156c-3p	177.95	115.48	87.47
miR156d-3p	0.55	0.74	0.28
miR156g-3p	0.01	0.01	0.09
miR156g-5p	23587.34	39991.51	27290.92
miR156h-5p	3252.14	3387.97	4061.82
miR156i-3p	4.07	5.17	4.17
miR156i-5p	51792.15	30261.08	19244.27
miR156j	14.41	9.6	6.31
miR159a	398.6	110.59	180.22
miR159b	0.01	0.01	8.9
miR160c	0.28	0.18	0.46
miR160e	10.63	11.45	30.05
miR164c	1155.87	695.19	1050.35
miR164d	0.55	0.09	0.46
miR166e-5p	39.55	26.49	12.71
miR166f	48.23	138.56	338.83
miR166g-3p	26405.96	15290.75	17745.45
miR166g-5p	234.41	139.39	59.83
miR167a	874.71	404.04	303.03
miR167b-5p	9207.97	4796.81	2322.76
miR168c-3p	113.83	99.6	55.19
miR168c-5p	4004.99	2690.11	2404.48
miR169e-5p	6.28	7.02	5.75
miR169f	0.37	0.09	0.09
miR169l-5p	0.65	0.18	0.46
miR171a	17.83	8.95	47.95
miR171b	0.28	0.18	0.37

miR171c	0.83	0.55	0.09
miR171d	1.39	1.85	1.67
miR171e-3p	0.55	0.65	1.11
miR171f	3.51	2.03	1.3
miR172a	38.71	11.35	7.88
miR172c-3p	7759.3	3361.57	2199.03
miR172c-5p	0.28	0.01	0.09
miR172d-3p	93.97	37.94	58.16
miR172d-5p	0.01	0.09	0.01
miR2089-5p	0.01	0.01	1.21
miR2118	179.99	119.73	225.67
miR2119	4.99	2.68	21.06
miR2199	552.34	342.93	685.92
miR2585d	22.27	26.86	27.36
miR2586a	7.67	10.06	9.28
miR2587g	0.28	0.37	0.28
miR2590j	318.86	419.64	260.83
miR2591	3.14	3.42	2.5
miR2592a-3p	0.09	0.09	0.09
miR2592am	5.91	4.71	3.9
miR2592bl-5p	0.09	0.09	0.09
miR2592bn-5p	4.8	3.88	3.34
miR2592bo-5p	0.28	0.18	0.19
miR2592br-3p	6.74	3.23	2.41
miR2592s-5p	0.46	0.46	0.28
miR2593d	0.37	0.01	0.37
miR2597	0.28	0.09	0.09
miR2598	1.02	2.49	1.3
miR2601	3.05	2.12	1.21
miR2603	1.57	1.57	1.11
miR2604	0.18	0.18	0.28
miR2606b	0.09	0.01	0.19
miR2606c	0.37	0.37	0.37
miR2608	1.2	1.02	1.02
miR2612	0.01	0.18	0.01
miR2614	1.48	1.29	1.02
miR2616	3.51	3.23	3.52
miR2625	0.37	0.09	0.09
miR2627	0.01	0.01	0.09
miR2629g	1.76	2.22	1.76
miR2630v	0.83	0.83	0.93
miR2638b	0.18	0.01	0.56
miR2645	0.46	0.37	0.28
miR2651	25.87	21.88	20.87

miR2652m	0.01	0.01	0.09
miR2655o	2.68	2.77	1.11
miR2666	2.59	2.86	2.23
miR2670d	0.18	0.09	0.09
miR2673b	0.74	0.01	0.93
miR2678	0.28	0.09	0.01
miR390	133.05	91.94	95.82
miR393b-5p	1.94	1.11	1.86
miR395o	0.28	0.18	0.09
miR396a-5p	174.07	52.06	35.15
miR396b-3p	61.35	37.39	27.36
miR396b-5p	136.65	99.79	139.32
miR397-5p	1.85	2.77	0.37
miR398a-3p	0.46	0.28	1.11
miR398a-5p	50.82	10.43	57.04
miR398c	2.59	0.74	0.37
miR399i	0.46	0.01	0.01
miR399t-3p	0.01	0.01	0.09
miR408-3p	3.88	1.85	3.25
miR408-5p	8.32	3.51	3.71
miR5037c	4.99	20.03	15.4
miR5205a	4.16	9.05	7.14
miR5205b	0.74	1.57	1.39
miR5205d	18.39	33.6	33.21
miR5207	2.03	3.97	4.17
miR5208c	1.66	3.97	2.32
miR5208d	0.83	0.55	1.02
miR5213-3p	0.01	0.09	0.01
miR5213-5p	173.7	112.06	156.66
miR5222	1.02	0.55	0.28
miR5228	0.18	0.18	0.46
miR5230	5.54	3.42	7.98
miR5231	1137.02	596.51	297.09
miR5232	29.66	31.85	14.75
miR5234	60.06	38.68	23.19
miR5237	9.7	6.09	8.63
miR5238	0.83	0.09	0.74
miR5239	1011.18	562.91	337.07
miR5241c	4.34	3.88	4.45
miR5242	0.46	0.09	0.19
miR5243	0.28	0.09	0.01
miR5244	10.53	7.85	10.95
miR5245	1.29	0.46	1.58
miR5248	11.27	9.51	7.7

miR5249	0.55	0.92	1.02
miR5254	0.09	0.01	0.01
miR5255	3.23	1.94	2.69
miR5256	0.18	0.18	0.09
miR5257	0.09	0.18	0.56
miR5261	2.59	1.85	1.76
miR5266	1.39	1.2	1.02
miR5267n	2.59	3.78	3.8
miR5267o	5.27	6.18	5.47
miR5269a	0.01	0.01	0.09
miR5269b	19.13	26.4	14.28
miR5272e	17.09	15.6	18.83
miR5272f	15.71	13.66	13.54
miR5273	3.97	4.06	6.03
miR5277	0.01	0.09	0.09
miR5279	12.2	22.62	16.23
miR5281a	3.14	5.26	5.01
miR5281f	57.38	97.11	86.82
miR5282	1.39	5.54	6.59
miR5283	0.46	0.92	0.37
miR5284b	2.22	3.97	1.11
miR5284g	115.03	154.71	100.08
miR5284h	0.01	0.09	0.01
miR5286a	12.2	13.66	14.56
miR5286b	15.52	16.25	7.7
miR5287a	25.32	33.23	30.24
miR5287b	170.75	138.93	47.95
miR5290	41.21	52.71	37.57
miR5291c	0.09	0.01	0.01
miR5292a	0.18	0.09	0.09
miR5294c	6.47	10.06	8.53
miR5295d	67.26	82.16	39.98
miR5297	8.96	7.48	4.73
miR5298c	18.94	34.43	22.17
miR5298d	5.91	8.03	4.73
miR5299	9.61	13.75	10.02
miR530	2.03	1.48	5.38
miR5561-3p	0.28	0.09	0.01
miR5741e	0.09	0.46	0.01
miR5743b	0.37	0.01	0.09
miR5744	0.65	0.37	0.28
miR5745a	0.28	0.18	0.09
miR5745b	0.46	0.09	0.37
miR5756	0.18	0.01	0.01

miR7696c-5p	0.28	0.18	0.01
miR7696d-3p	7.21	4.71	4.82
miR7696d-5p	0.01	0.09	0.01
miR7699-5p	0.09	0.09	0.37
miR7701-3p	0.28	0.18	0.09
miR7701-5p	0.65	0.46	0.28
NmiR0005	19.03	12.09	16.42
NmiR0006	1.11	1.48	0.09
NmiR0008	43.33	33.14	27.46
NmiR0009	11.27	15.79	8.81
NmiR0015	62.46	68.03	58.53
NmiR0018	381.87	190.07	314.07
NmiR0019	772.43	504.2	63.54
NmiR0022	15.89	13.85	14.01
NmiR0026	1263.6	785.84	489.28
NmiR0027	3.51	2.31	1.58
NmiR0028	73.55	41.08	35.06
NmiR0029	100.06	50.86	27.73
NmiR0033	1496.07	1053.81	972.63
NmiR0037	53.5	56.77	70.31
NmiR0041	24.12	21.05	28.38
NmiR0043	16.45	11.54	4.36
NmiR0047	99.33	72.93	21.89
NmiR0049	6988.44	2023.26	1166.48
NmiR0051	2487.11	604.17	536.59
NmiR0053	11.92	8.31	2.6
NmiR0057	1216.57	1402.47	1435.56
NmiR0058	0.46	0.46	0.09
NmiR0062	6.74	4.06	4.27
NmiR0063	6.01	4.43	3.8
